# HDAC3-YY1-RAB5A axis remodels AML-supportive niche by modulating mitochondrial homeostasis in bone marrow stromal cells

**DOI:** 10.1038/s41419-025-07777-9

**Published:** 2025-07-07

**Authors:** Chao He, Yue Xiong, Yuqing Zeng, Jianhua Feng, Fuxia Yan, Manqi Zhang, Zhili Tan, Yaling Zheng, Hongbo Chen, Rui Huang, Fang Cheng

**Affiliations:** 1 School of Pharmaceutical Sciences (Shenzhen), Sun Yat-sen University, Shenzhen, China; 2 Institute of Chemical Biology, Shenzhen Bay Laboratory, Shenzhen, China; 3 The Eighth Affiliated Hospital, Sun Yat-sen University, Shenzhen, China; 4 Department of Hematology, Zhujiang Hospital of Southern Medical University, Guangzhou, China; 5 The Affiliated Dongguan Songshan Lake Central Hospital, Guangdong Medical University, Dongguan, Guangdong China

**Keywords:** Cancer microenvironment, Acute myeloid leukaemia

## Abstract

Recent studies have shown that the interaction between acute myeloid leukemia (AML) and bone marrow stromal cells (BMSCs) plays a vital role in the progression of leukemia and the development of drug resistance, while the underlying mechanisms remain inconclusive. In this study, we found that AML patient-derived BMSCs exhibit a hyperinflammatory phenotype. Histone deacetylase 3 (HDAC3) in BMSCs enhances mitochondrial reactive oxygen species (ROS) production by RAB5A-mediated blockade of mitophagy. Furthermore, we confirmed that HDAC3 regulates RAB5A expression through transcription factor YY1. Excessive ROS accelerates the senescence of BMSCs and promotes the secretion of senescence-associated secretory phenotype, creating a hyperinflammatory bone marrow niche, activating the NF-κB pathway in AML cells to promote their survival and drug resistance. The inhibition of HDAC3 in BMSCs reduces the mitochondrial ROS production and thus delays BMSCs senescence. Consequently, HDAC3 inhibition in BMSCs decreases AML proliferation and synergizes with the anti-AML efficacy of venetoclax. Therefore, our study suggests that targeting HDAC3 in BMSCs may be used for the combination therapy of AML by remodeling the AML-supportive niche.

## Highlights

- HDAC3-induced mitochondrial oxidative stress contributes to the formation of inflammatory BMSCs.
- The HDAC3-YY1-RAB5A signaling pathway is involved in the regulation of mitochondrial homeostasis.
- Inhibition of HDAC3 attenuates the mitophagy and inflammatory response of AML-BMSCs.
- Inhibition of HDAC3 blocks the progression of AML induced by AML-BMSCs.

## Graphical abstract

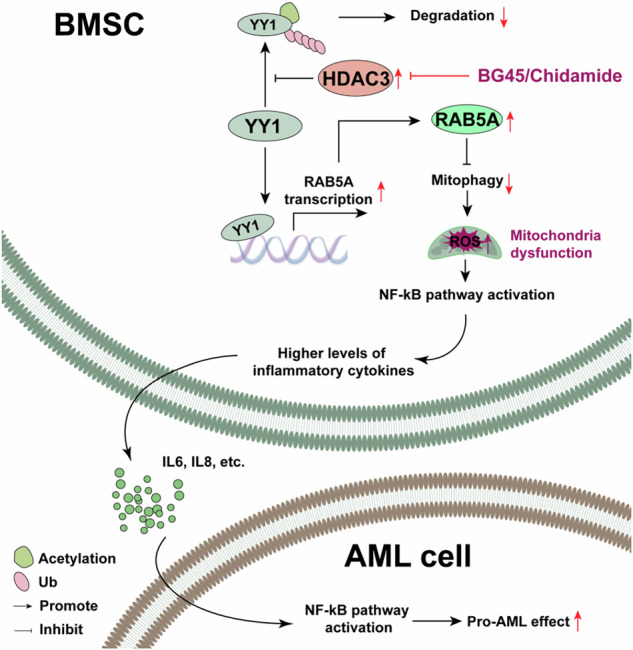

## Introduction

Acute myeloid leukemia (AML) is a highly lethal blood cancer associated with a chronic inflammatory environment in the bone marrow [1–3]. Emerging evidence strongly suggests that the complex interplay between tumor cells and their micro-environment can contribute to the failure of antitumor therapy [4–6]. Similarly, during leukemogenesis, AML cells progressively reshape other resident cells, especially bone marrow stromal cells (BMSCs), in the bone marrow niche (BMN) and hijack them into leukemia-supportive cells through direct contact or paracrine secretion [7, 8]. Interestingly, AML cells prefer to use more oxidative phosphorylation than glycolysis to escape chemotherapy [9–11]. However, this reliance on oxidative metabolism necessitates a higher number of mitochondria to meet the substantial energy demands of cancer cells. Superoxide generated by AML cells can be passed to BMSCs and stimulate the transfer of mitochondria through AML-derived tunneling nanotubes to AML cells, thus providing extra mitochondria for the oxidative phosphorylation [12]. The depletion of Nestin^+^ BMSCs contribute to slowing leukemia progression in primary AML mice and reducing AML cells in chimeric mice through blocking co-opt energy sources and antioxidant defense mechanisms from BMSCs [13]. Additionally, exosomes derived from AML cells increase mesenchymal stromal progenitors and suppress osteogenesis, resulting in the damage of normal hematopoiesis [7]. In turn, BMSCs are propelled by exosomes released from AML cells to generate IL8 and protect them from chemotherapy drug induced apoptosis [14]. Analysis of the bone marrow micro-environment cells of AML patients using single-cell sequencing revealed that LEPR^+^ BMSCs exhibited a high inflammatory phenotype, which inhibited normal hematopoietic cell function and promoted the survival of AML under chemotherapy [15]. Proinflammatory factors have been widely shown to be involved in the progression of AML. For example, IL6 promotes chemotherapy resistance of AML cells through mitochondrial fusion mediated by Mitofusin-1 [16], and the IL6 level of patients can predict the progression-free survival of children with AML [17]. BMSCs are also IL6 secreting cells, and studies have shown that IL6 derived from AML-BMSCs can promote chemotherapy resistance and epithelial-mesenchymal transition (EMT) in AML through the IL6-STAT2/3 signaling pathway [18]. However, the intrinsic molecular mechanism of the formation of the inflammatory phenotype of BMSCs has not yet been fully revealed.

Histone deacetylases are a class of epigenetic regulatory elements that regulate protein acetylation levels [19]. In the mammalian genome, the HDAC superfamily consists of 11 HDAC subtypes, which are involved in many physiological activities such as cell growth, differentiation, and apoptosis [20, 21]. HDAC3 is a distinct subtype of HDACs that exerts its catalytic activity by forming a complex with the nuclear receptor corepressor 1 (NCoR1) and the silencing mediator of retinoic acid and thyroid hormone receptors (SMRT) [22]. Beyond this, HDAC3 also has significant non-enzymatic roles independent of these two nuclear receptor corepressors [23]. Growing evidence indicates that HDAC3 plays a crucial role in physiological processes, including the regulation of mitochondrial metabolism [24], modification of antioxidant genes, and sensitivity to reactive oxygen species (ROS). The inhibition of HDAC3 stabilizes NRF2 and helps to alleviate the oxidative stress damage of endothelial cell induced by type 2 diabetes mellitus [25]. HDAC inhibitors (HDACi) have also been widely studied in the treatment of AML [26, 27], among which chidamide, as the first oral inhibitor, has received increasing attention [28, 29]. Chidamide has been used in combination with venetoclax and azacitidine to treat venetoclax-resistant AML (NCT05566054), demonstrating promising therapeutic effects in clinical studies [30]. Given the role of HDACi in the regulation of mitochondrial function and oxidative stress, we speculate that in addition to its direct anti-tumor effect, it may also have the potential to regulate the phenotype of BMSCs. However, the role of HDAC in the formation of the inflammatory phenotype of BMSCs has not been confirmed.

In our study, we point out the role of HDAC3 in the inflammatory phenotype of AML-BMSCs. Mechanistically, AML cell secretomes inhibit mitophagy and elevate mitochondrial reactive oxygen species (ROS) production by regulating the HDAC3-YY1-RAB5A signaling pathway in BMSCs. The resulting ROS accumulation accelerates BMSC senescence and promotes the release of pro-inflammatory cytokines associated with the senescence-associated secretory phenotype (SASP). This contributes to the formation of a hyperinflammatory bone marrow micro-environment, which activates the NF-κB pathway in AML cells and enhances their survival and drug resistance. Conversely, inhibiting HDAC3 in BMSCs reduces mitochondrial ROS production, thereby delaying the senescence of these cells. Therefore, our research introduces a novel strategy to effectively address the therapeutic challenges associated with the AML bone marrow micro-environment.

## Results

### AML patient-derived BMSCs exhibits a hyperinflammatory phenotype

**Fig. 1:**
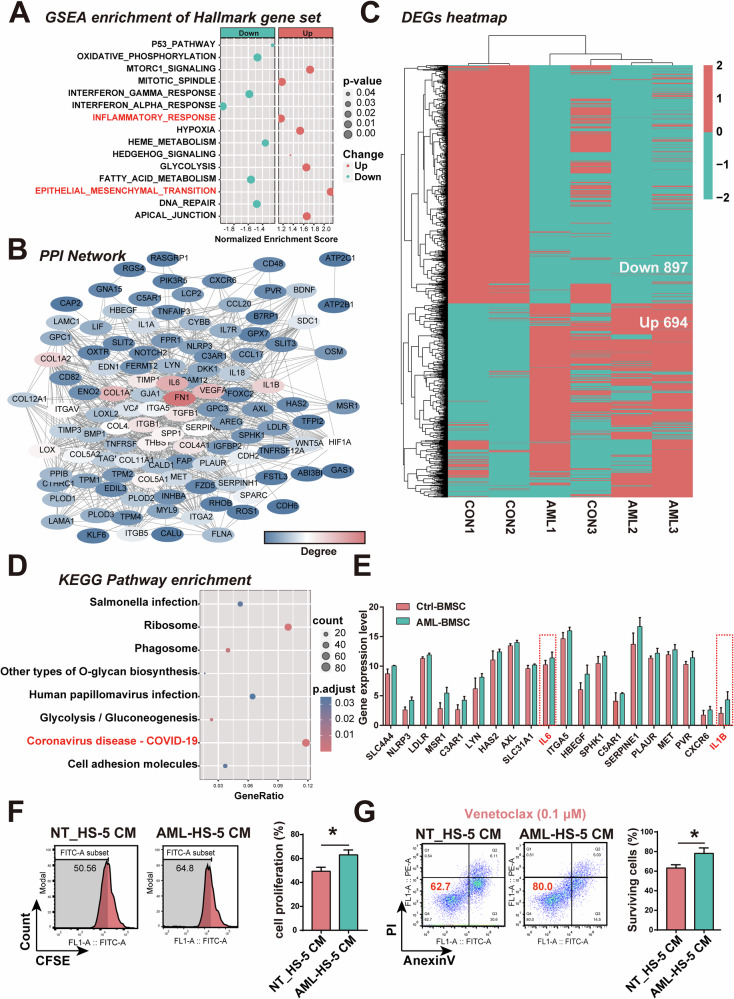
Gene expression profile of BMSCs derived from AML patients. **A** GESA enrichment plot of expression data from AML patients derived BMSCs (GSE107490) based on Hallmark gene sets. **B** Protein-protein interaction network of enriched genes from EMT and inflammatory response gene set. **C** Heatmap of differentially expressed genes in AML-BMSCs compared to control healthy-BMSCs. **D** KEGG pathway enrichment plot. **E** Expression level of genes involved in inflammatory response. **F** The proliferation of primary AML cells treated by conditioned medium from nontreated (NT_HS-5-CM) or AML-treated HS-5 (AML-HS-5 CM). **G** The apoptosis of primary AML cells treated by venetoclax in the presence of conditioned medium from nontreated (NT_HS-5-CM) or AML-treated HS-5 (AML-HS-5 CM). Data are presented as the mean ± SD. *n* = 3, *p < 0.05.

Bone marrow stromal cells (BMSCs) in the local bone marrow micro-environment play a critical role in supporting AML onset and progression. To better understand the impact of the bone marrow niche on AML pathogenesis, we analyzed the global transcriptome of BMSCs isolated from human AML patients (AML-BMSCs) and healthy bone marrow donors using data from the Gene Expression Omnibus (GEO) database (GSE107490). Gene set enrichment analysis (GSEA) revealed that AML-BMSCs exhibited a significant enrichment of gene signatures related to multiple signaling pathways including epithelial-mesenchymal transition and inflammatory responses (Fig. 1A). Inflammatory response-related genes, particularly IL6 and IL1B, were identified as key regulators in the protein-protein interaction (PPI) network of enriched genes from above two pathways (Fig. 1B). Furthermore, using DESeq2, we identified 694 upregulated and 897 downregulated genes in AML-BMSCs (Fig. 1C). KEGG pathway analysis highlighted strong enrichment in genes related to the COVID-19 hyperinflammatory signaling pathway (Fig. 1D). Specifically, the expression of genes such as IL6 and IL1B was elevated in AML-BMSCs in the inflammatory response gene set (Fig. 1E). We then tested the IL6 and IL1B levels of AML-BMSCs. The results showed that the IL6 and IL1B levels of induced AML-BMSCs were significantly increased (Fig. S1A). Similarly, in remission and relapse patients, it was found that BMSCs from relapsed/refractory (R/R) patients had higher IL6 and IL1B levels (Fig. S1B, C). Interestingly, previous studies have also shown that IL6 and IL1B promote AML cell proliferation [31, 32] and contribute to drug resistance [17, 33]. Additionally, AML-BMSCs secretions were found to enhance the proliferation of primary AML cells and protect them from venetoclax-induced apoptosis (Fig. 1F, G and Fig. S1D). Collectively, these findings demonstrate that AML-BMSCs contribute to a hyperinflammatory micro-environment, and modulating their inflammatory phenotype may aid in developing effective anti-AML therapies.

### AML-induced upregulation of HDAC3 in BMSCs modulates the inflammatory cellular phenotype

**Fig. 2:**
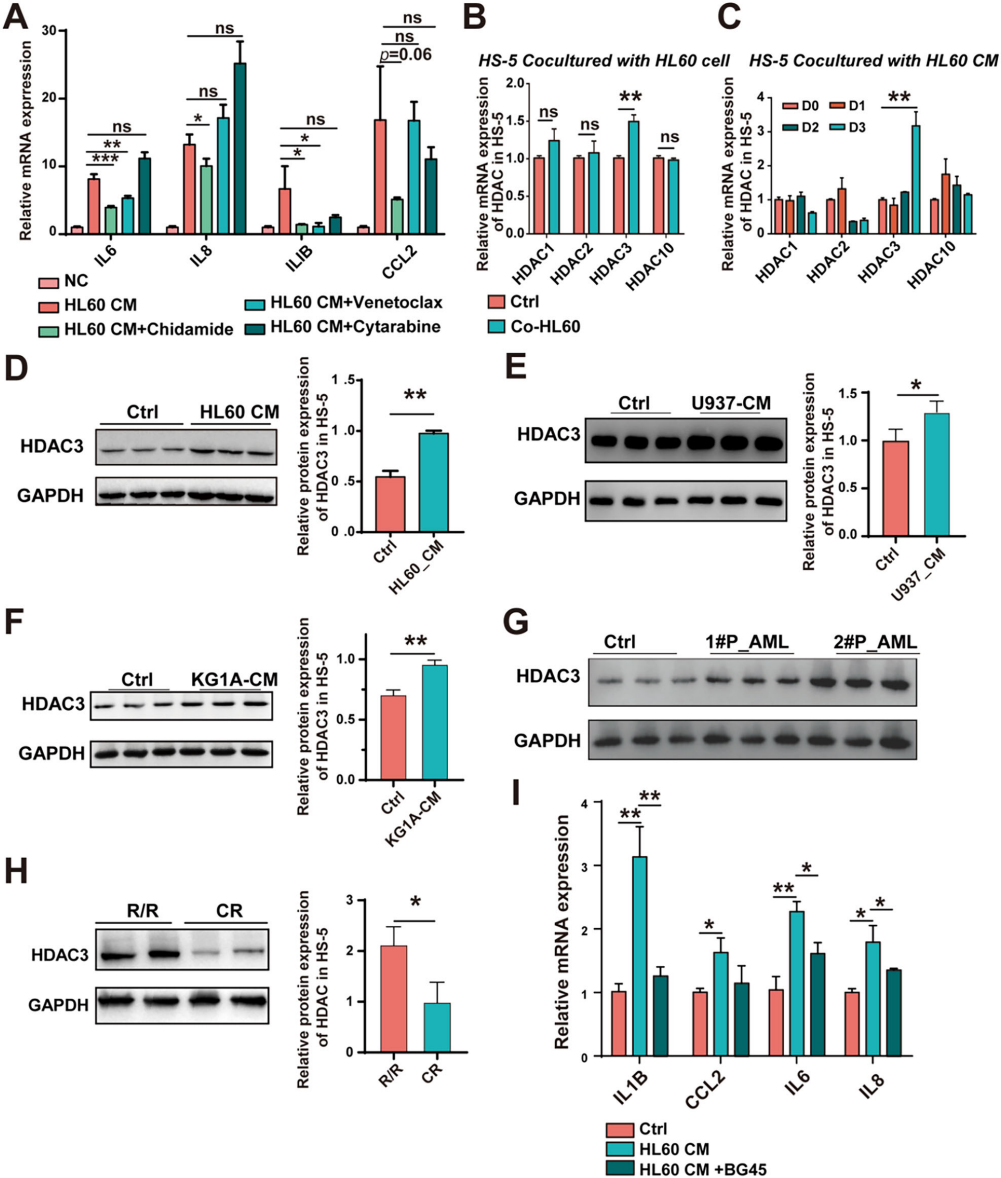
HDAC3 in BMSCs modulates the hyperinflammatory phenotype. **A** mRNA expression of IL6, IL8, IL1B and CCL2 in HS-5. **B**, **C** mRNA expression of HDACs in HS-5 cocultured with HL60 directly or indirectly, respectively. **D–G** Protein expression of HDAC3 in HS-5 after being cultured with AML cell lines or AML patient cells-derived conditioned medium. **H** Protein expression of HDAC3 in BMSCs from RR (refractory or relapsed) or CR (complete remission) AML patients. **I** mRNA expression of IL6, IL8, IL1B and CCL2 in HS-5.All blots were normalized with a loading control. Data are presented as the mean ± SD. *n* = 3, *p < 0.05, **p < 0.01, ***p < 0.001.

As developing therapeutics simultaneously targeting both tumor cells and the surrounding micro-environment offer dual therapeutic advantages, we analyzed the effects of current clinically used AML drugs in modulating the hyperinflammatory bone marrow niche. First, we established a cell model by using conditioned medium (CM) from an AML cell line, HL60 to induce inflammatory cytokines in HS-5 bone marrow stromal cells, including IL6, IL1B, IL8 and CCL2 (Fig. 2A). Three existing AML treatments: cytarabine (a cytotoxic agent), venetoclax (a BCL-2 inhibitor), and chidamide (a histone deacetylase inhibitor) were examined on affecting the inflammatory phenotype of BMSCs. Among the drugs tested, chidamide significantly inhibited the mRNA expression of these cytokines in BMSCs (Fig. 2A). As a pan-HDAC inhibitor, chidamide targets subtypes 1, 2, and 3 of class I HDAC and subtype 10 of class IIb. To identify the specific subtype responsible for reversing the hyperinflammatory phenotype of BMSCs, we first cultured HL60 cells on a monolayer of HS-5 stromal cells, and there was a significant upregulation (1.5-fold) of HDAC3 expression in co-culture HS-5 cells (Fig. 2B). Similarly, co-culture with AML cells derived CM also induced HDAC3 expression in HS-5 cells (Fig. 2C and Fig. S2A–C). Western blot analysis further confirmed that the treatment with the CM from HL60 as well as AML cell lines (U397 and KG1A) and primary AML cells increased HDAC3 protein levels in HS-5 cells (Fig. 2D–G and Fig. S2D). In line with these findings, BMSCs freshly isolated from primary AML cells displayed elevated HDAC3 expression in R/R patients compared to those patiens in complete remission (CR) (Fig. 2H). Moreover, pharmacological inhibition of HDAC3 in HS-5 cells using the BG45 reversed AML cells’ CM-induced mRNAs expression of inflammatory cytokines (Fig. 2I and Fig. S2E, F). However, the use of CD34^+^ cell-derived CM did not significantly affect the inflammatory factors as well as the mRNA levels of HDAC3 (Fig. S2G). Collectively, these results suggest that targeting HDAC3 in BMSCs could mitigate the inflammatory cellular phenotype.

### HDAC3 plays a crucial role in regulating the homeostasis of mitochondrial oxidative stress in BMSCs

**Fig. 3:**
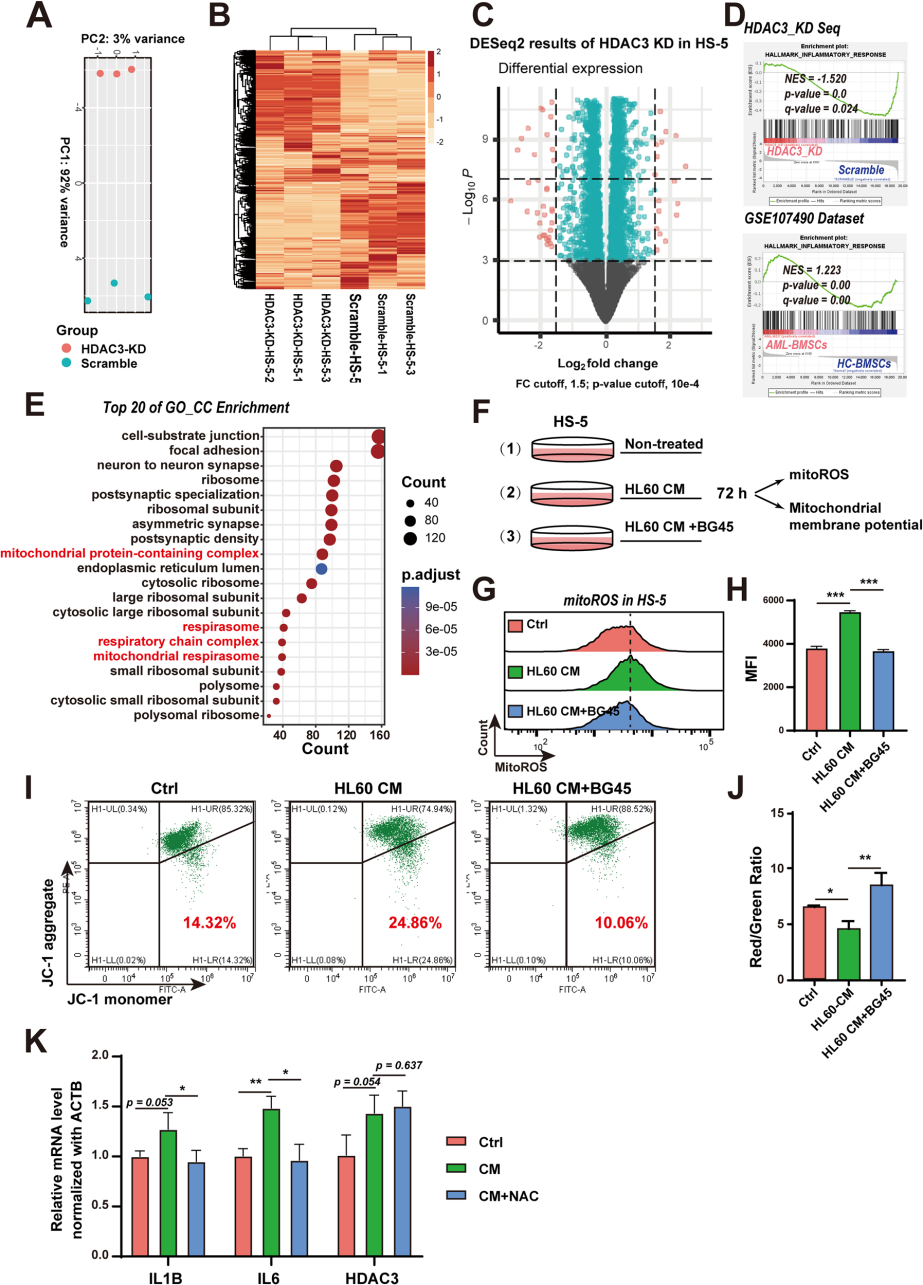
HDAC3 plays a crucial role in regulating the homeostasis of mitochondrial oxidative stress in BMSCs. **A** PCA analysis plot. **B** Expression heatmap of differentially expressed genes. **C** volcano plot of all detected genes. **D** GSEA result showed inflammatory response gene set was significantly enriched both in shHDAC3 sequencing and GSE107490. **E** GO cellular component enrichment (GO_CC) analysis of genes downregluated in shHDAC3 group. **F** Schematic diagram of experimental flow. **G, H** Flow cytometry analysis of ROS level in HS-5 treated by Ctrl, HL60 CM, HL60 CM + BG45 after 72 h. The right panel is the statistics of mean fluorescence intensity. **I, J** Flow cytometry analysis of JC-1 fluorescence in HS-5 treated by Ctrl, HL60 CM, HL60 CM + BG45 after 72 h. **K** mRNA level of IL6, IL1B and HDAC3 in HS-5 cells induced by AML cells derived CM and CM + NAC. Data are presented as the mean ± SD. *n* = 3, *p < 0.05, **p < 0.01, **p < 0.001.

Having demonstrated that HDAC3 inhibition significantly down-regulates pro-inflammatory cytokine expression in BMSCs, we aimed to elucidate the molecular mechanisms by which HDAC3 in BMSCs regulates the hyperinflammatory environment in AML. We first established an HDAC3 knockdown HS-5 cell line. We then examined the expression levels of inflammatory factors in shHDAC3 HS-5 cells before and after CM treatment and found that HDAC3 knockdown significantly inhibited inflammatory cytokine expression (Fig. S2H). Next, we performed a global transcriptome analysis to compare differentially expressed genes between scramble control and shHDAC3 HS-5 cells. Principal component analysis (PCA) of mRNA expression data revealed a distinct gene expression profile between the two groups (Fig. 3A). A total of 19,225 genes were detected, with 857 upregulated and 734 downregulated in shHDAC3 HS-5 cells compared to the scramble group (padj < 0.01, |log2FC | > 0.5) (Fig. 3B-C). Gene set enrichment analysis (GSEA) provided further insight into the signaling networks modulated by HDAC3. The inflammatory response, which was previously activated in dataset GSE107490, was significantly suppressed in shHDAC3 HS-5 cells (Fig. 3D), further indicating the contribution of HDAC3 to the formation of inflammatory phenotype of BMSCs.

However, the activation of inflammatory response pathways is often a secondary response of cells, prompting the need to investigate how HDAC3 regulates inflammatory signals. Through KEGG pathway enrichment analysis, we identified several key pathways involved in the HDAC3 regulatory network. In addition to inflammatory regulation (e.g., coronavirus disease-COVID-19), pathways related to neurodegenerative diseases, reactive oxygen species (ROS), and oxidative phosphorylation were significantly enriched (Fig. S3A). Gene Ontology cellular component (GO_CC) analysis further revealed that HDAC3 may serve as a key regulator of mitochondrial respiration (Fig. 3E). These bioinformatics findings suggest that HDAC3 could regulate mitochondrial oxidative stress in BMSCs. Therefore, we assessed mitochondrial quality in BMSCs upon different treatments (Fig. 3F). It was observed that the incubation with AML CM promoted the generation of mitochondrial ROS (mitoROS) in HS-5 cells, which was reversed by HDAC3 inhibitor BG45 (Fig. 3G, H). Downregulation of HDAC3 in BMSCs reduced mitoROS accumulation (Fig. S3B, C). Furthermore, the mitochondrial membrane potential damaged by AML CM was also restored by BG45 (Fig. 3I, J and Fig. S3D). Additionally, treatment with the ROS scavenger NAC mitigated ROS-induced damage and reduced the CM-induced upregulation of inflammatory cytokine mRNA levels (Fig. 3K). Collectively, these results demonstrate that HDAC3 plays a crucial role in regulating the homeostasis of mitochondrial oxidative stress in BMSCs.

### HDAC3 inhibits mitophagy in BMSCs by increasing RAB5A expression

**Fig. 4:**
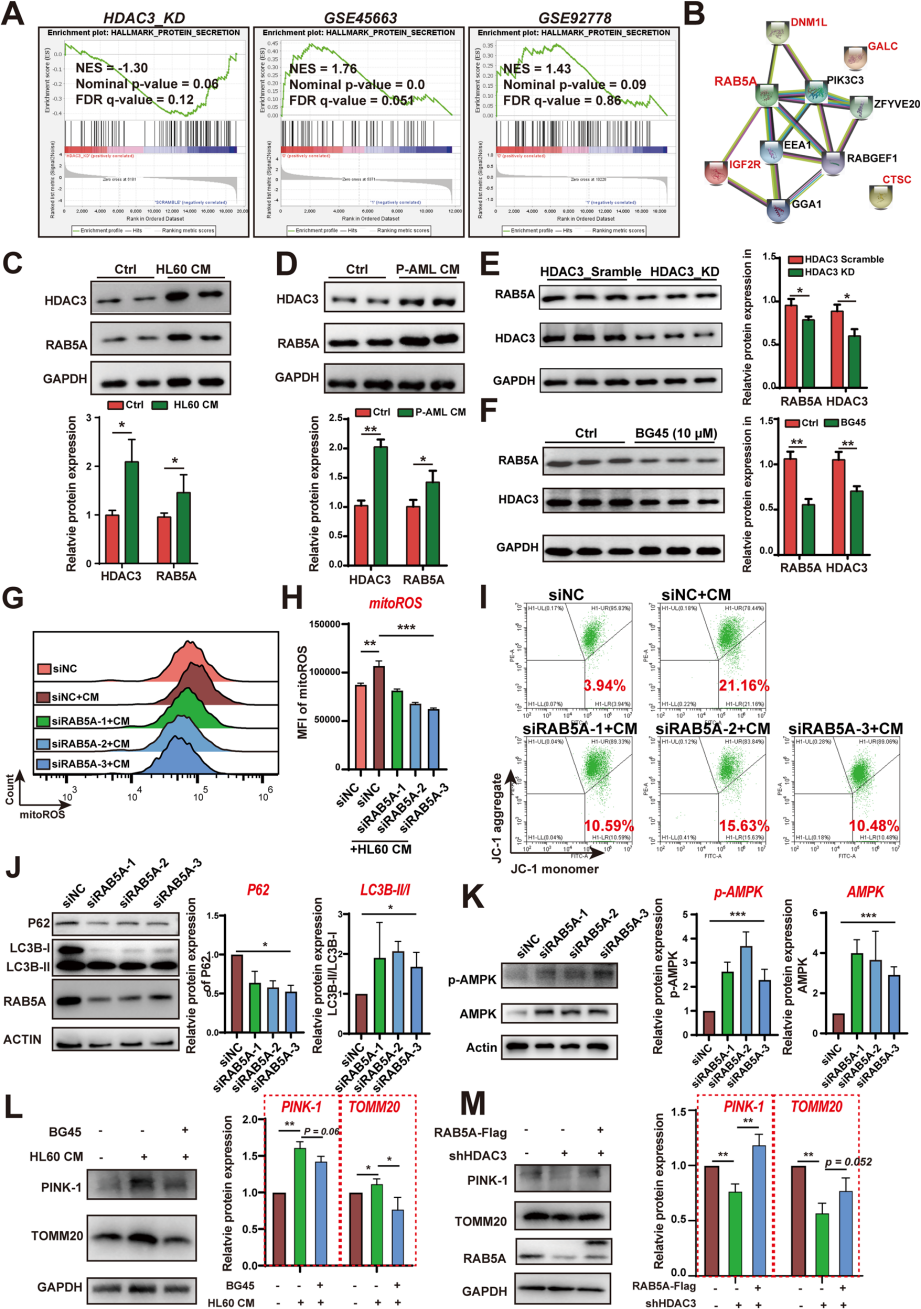
HDAC3 inhibition reduces the mitochondrial ROS production by regulating RAB5A. **A** GSEA enrichment analysis showed a significant increase in the enrichment of the protein secretion gene set in the shHDAC3, GSE45663, and GSE92778 datasets. **B** Protein-protein interaction network diagram of intersected genes and their associated proteins. **C, D** Immunoblot and quantitative analysis of RAB5A and HDAC3 protein expression in HS-5 cells treated with HL60 or primary AML patient-derived CM. **E**, **F** Immunoblot and quantitative analysis of RAB5A and HDAC3 protein expression in HS-5 cells treated with shHDAC3 vector or BG45. **G**, **H** Flow cytometry analysis of mitoROS level in HS-5 treated by siNC, siRAB5A-1, siRAB5A-2 and siRAB5A-3 after 72 h. The right panel is the statistics of mean fluorescence intensity. **I** Flow cytometry analysis of JC-1 fluorescence in HS-5 treated by siNC, siRAB5A-1, siRAB5A-2 and siRAB5A-3 after 72 h. **J**, **K** Representative immunoblot images of p62, LC3B, AMPK and p-AMPK expression in siNC, siRAB5A-1, siRAB5A-2 and siRAB5A-3 HS-5 cells. The right panel is the statistic result. The data was normalized to loading control. **L**, **M** Representative immunoblot images showing PINK-1 and TOMM20 expression in HS-5 cells under the indicated treatment conditions. All blots were normalized with loading control. Data are presented as the mean ± SD. *n* = 3, *p < 0.05, **p < 0.01.

As an epigenetic regulator, HDAC3 is widely involved in multiple physiological activities of cells. To systematically elucidate the mechanism by which HDAC3 regulates mitochondrial oxidative stress homeostasis in BMSCs, we compared the sequencing data of shHDAC3-BMSCs with transcriptome data from patient-derived BMSCs (GSE92778) and AML cell co-culture-treated BMSCs (GSE45663) using Gene Set Enrichment Analysis (GSEA). In addition to the inflammatory response gene set, the protein secretion gene set was significantly enriched across these three datasets (Fig. 4A and Fig. S4A). Further analysis revealed five co-enriched genes: RAB5A, IGF2R, DNM1L, GALC, and CTSC, with RAB5A at the core of the regulatory network, as indicated by protein interaction network analysis (Fig. 4B and Fig. S4B). Notably, gene correlation analysis using GEPIA2 showed that HDAC3 expression was most strongly associated with RAB5A (R = 0.78, *p*-value = 0) compared to the other four co-enriched genes (Fig. S4C). Both AML primary cell culture supernatant and AML cell line-derived conditioned media (CM) can simultaneously induce HDAC3 and RAB5A expression in BMSCs (Fig. 4C, D, Fig. S4D). Furthermore, pharmacological or genetic inhibition of HDAC3 downregulated RAB5A expression (Fig. 4E, F). These results collectively suggest that HDAC3 positively regulates RAB5A expression in BMSCs.

Next, we considered RAB5A as a hub gene and constructed a RAB5A interactome. As shown in Fig. S4E, the enrichment analysis indicated that RAB5A may be involved in mitochondrial depolarization, mitophagy, and peroxisome autophagy. This suggests that HDAC3 may regulate mitochondrial homeostasis *via* RAB5A. To test this hypothesis, we first assessed mitochondrial ROS levels in siRAB5A HS-5 cells. Consistently, flow cytometry analysis revealed a significant reduction in mitochondrial ROS levels in siHDAC3 HS-5 cells compared to siNC controls (Fig. 4G, H). Additionally, RAB5A silencing in HS-5 cells partially restored the damage to mitochondrial membrane potential caused by AML CM (Fig. 4I).

Analysis of the RAB5A interactome revealed its potential role in regulating mitophagy. Therefore, we investigated the function of the HDAC3-RAB5A axis in autophagy flux formation in BMSCs. We found that PE-labeled LC3B (LC3B-II, a major marker of autophagic activity) expression was significantly downregulated in the presence of HL60 conditioned media (CM) (Fig. S5A). Immunofluorescence analysis of another autophagy marker p62 demonstrated its accumulation in HS-5 cells following HL60 CM treatment, which was reversed by the treatment of BG45 (Fig. S5B, C). Using LC3B-RFP-GFP to track autophagosome-lysosome fusion, we observed increased green fluorescence in LC3B after HL60 CM treatment, indicating a block in autophagosome-lysosome fusion that was rescued by BG45 (Fig. S5D). Genetic inhibition of HDAC3 resulted in the upregulation of LC3B-II expression in HS-5 cells (Fig. S5E). These findings suggest that AML downregulates autophagic activities in BMSCs, which can be reversed by HDAC3 inhibition. When we silenced RAB5A expression using siRNA, we observed an increase in LC3B-II and a decrease in total p62 protein, indicating that RAB5A knockdown promotes autophagy in BMSCs (Fig. 4J). Additionally, we assessed the total protein and phosphorylation levels of AMPK, which are associated with mitochondrial autophagy. RAB5A silencing promoted an increase in AMPK protein level as well as it phosphorylation (Fig. 4K). Furthermore, we examined changes in TOMM20 and PINK1 levels in HS-5 cells following HL60 CM treatment and found that HL60 CM exposure simultaneously increased PINK1 and TOMM20 levels. This increase may be associated with the accumulation of damaged mitochondria due to autophagic flux blockade. Upon addition of BG45 to inhibit HDAC3, the levels of both PINK1 and TOMM20 were restored (Fig. 4L). To further investigate the role of RAB5A, we complemented HDAC3-knockdown HS-5 cells with RAB5A-Flag and observed that HDAC3 knockdown reduced RAB5A expression along with a decrease in PINK1 levels (Fig. 4M). Notably, RAB5A complementation upregulated PINK1 levels (Fig. 4M), providing additional evidence that RAB5A is involved in the regulation of mitophagy. Collectively, these indicate that HDAC3 plays a crucial role in regulating mitochondrial oxidative stress and mitophagy in BMSCs through RAB5A.

**Fig. 5:**
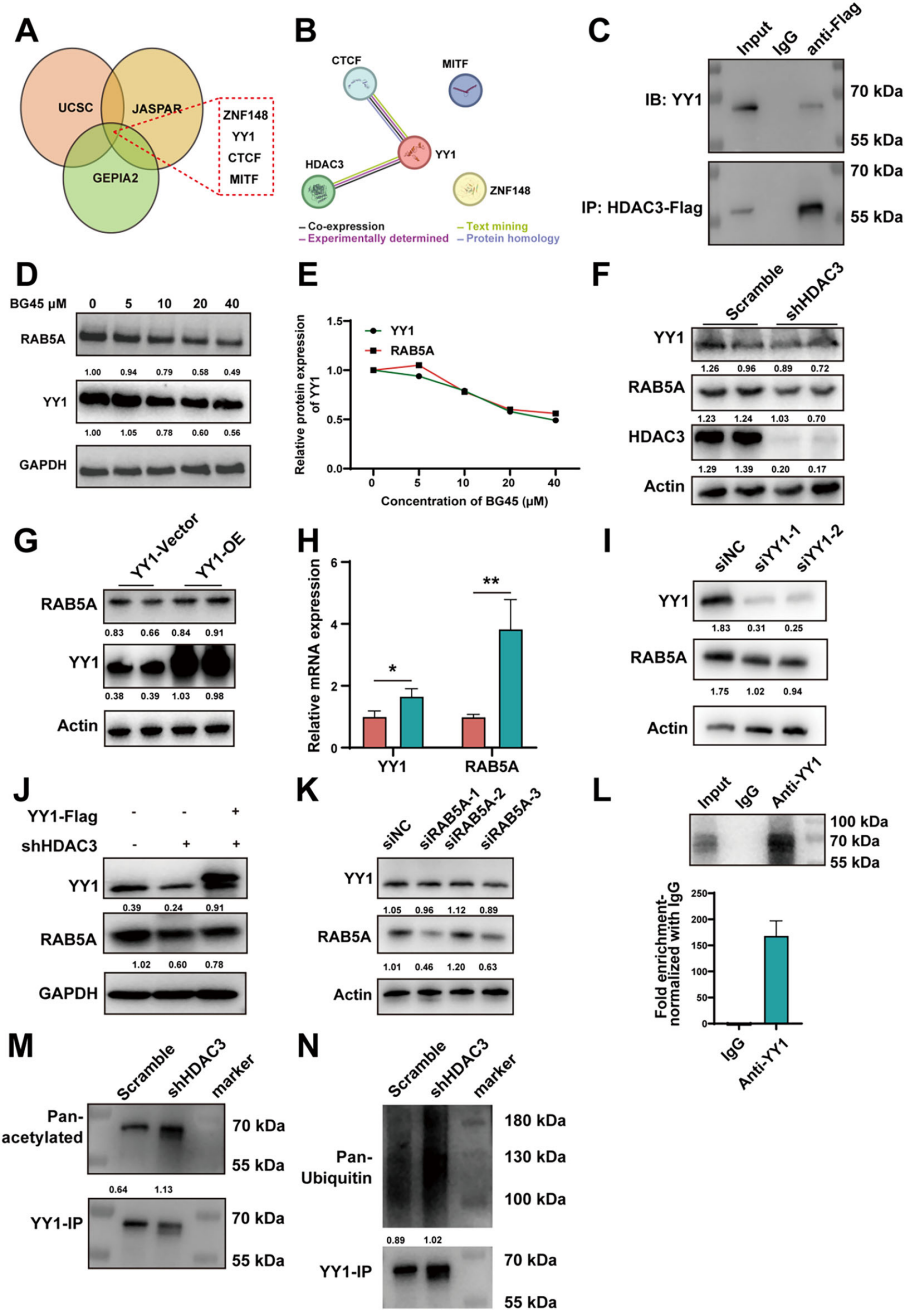
HDAC3 inhibits YY1 acetylation to modulate RAB5A. **A** UCSC, JASPAR and GEPIA2 databases were used to search for transcription factors that regulate RAB5A. **B** Candidate transcription factors interacting with HDAC3 were identified based on the STRING database (https://cn.string-db.org/). **C** HDAC3-flag was pulled down using anti-Flag antibody, and YY1 in the precipitated proteins was detected by immunoblotting. **D** Relative protein expression level of YY1 and RAB5A in HS-5 cells following treatment with varying concentrations of BG45. **E** Quantification of **D**. **F** Immunoblots of YY1 and RAB5A after knocking down HDAC3. **G** Immunoblots of RAB5A after YY1 overexpression. **H** Changes in the level of RAB5A mRNA after overexpressing YY1. **I** Immunoblots of RAB5A after knocking down YY1. **J** Immunoblots of RAB5A after complementation with YY1-Flag. **K** Immunoblots of YY1 expression after knocking down RAB5A. **L** Relative enrichment of YY1 at RAB5A promoter assessed by ChIP-qPCR. YY1 was pulled down using YY1 antibody, and the acetylation (**M**) and ubiquitination level (**N**) of YY1 after HDAC3 knockdown was detected by immunoblotting. All blots were normalized with a loading control. Data are presented as the mean ± SD. *n* = 3, *p < 0.05, **p < 0.01.

### HDAC3 inhibits YY1 acetylation to modulate RAB5A

Next, we tried to find the intrinsic mechanism of HDAC3 regulating RAB5A. Our results showed that HDAC3 can positively regulate RAB5A transcription and consequent protein expression, so we speculated that HDAC3 may affect the transcription factors that regulate the transcription of RAB5A. Through predictions based on the UCSC, GEPIA2 and JASPAR databases, we found that four genes including YY1 may be transcriptional regulators of RAB5A (Fig. 5A). Based on the STRING database, we found that YY1 interactes with HDAC3 (Fig. 5B), so we reasonably speculated that HDAC3 may regulate the expression of RAB5A through YY1. Through immunoprecipitation, we detected that HDAC3 interacted with YY1 (Fig. 5C), and inhibiting the expression of HDAC3 by BG45 could simultaneously inhibit the levels of YY1 and RAB5A (Fig. 5D, E and Fig. S6A, B). Knocking down the expression of HDAC3 at the genetic level also downregulated the levels of YY1 and RAB5A (Fig. 5F). Next, we constructed YY1 overexpression and knockdown cell lines. As shown in Fig. 5G–I, the enhancement of YY1 expression promoted the RAB5A expression, whereas YY1 knockdown downregulated RAB5A expression. Furthermore, complementation of shHDAC3 HS-5 cells with YY1-Flag restored RAB5A expression (Fig. 5J). Notably, siRNA-mediated knockdown of RAB5A did not affect YY1 expression levels (Fig. 5K). ChIP-PCR further confirmed that YY1 functions as a transcriptional regulator of RAB5A expression (Fig. 5L and Fig. S6C), indicating that YY1 plays a role in regulating RAB5A transcription. Previous study have shown that the stability of YY1 is regulated by its acetylation and ubiquitination levels [34]. Therefore, we tested the effect of HDAC3 on the acetylation level of YY1. Our findings indicate that HDAC3 knockdown promoted YY1 acetylation while enhancing its ubiquitination (Fig. 5M, N). Collectively, these results suggest that HDAC3 stabilizes YY1 by modulating its ubiquitination, thereby regulating RAB5A expression.

**Fig. 6:**
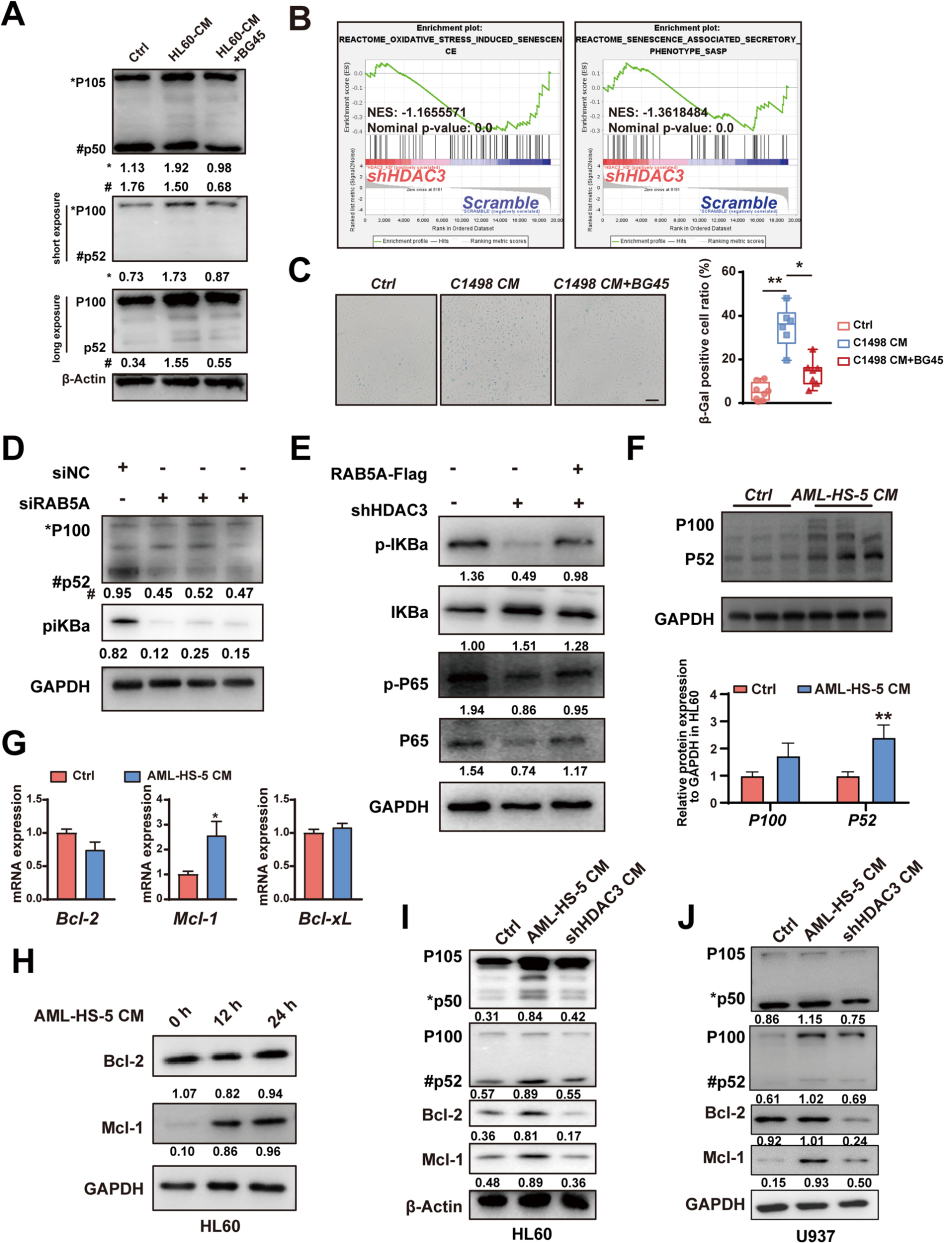
HDAC3 inhibition delays cellular senescence of BMSCs and downregulated NFKB-MCL1 pathway in AML. **A** Representative immunoblotting image of NFKB1 and NFKB2 in HS-5 treated with Ctrl, HL60 CM, and HL60 CM + BG45. **B** GSEA was performed on senescence-related gene sets. **C** The left panel showed the representative image of β-Gal staining of primary BMSCs derived from C57BL/6J treated with Ctrl, C1498 CM, and C1498 CM + BG45; the right panel showed the statistical image of β-Gal positive cells. Scale bar =100 μm. **D** Representative immunoblotting image of NFKB2 (P100/P52), IKBα, and pIKBα in HS-5 cells transfected by siNC and siRAB5A. **E** Immunoblotting image of NF-KB pathway-related proteins expression in HS-5 cells. **F** Representative immunoblotting image of NFKB2 in HL60 treated with Ctrl and AML-HS-5 CM. **G** The mRNA expression of BCL2, MCL1, and BCLXL in HL60 treated with Ctrl and AML-HS-5 CM. **H** Representative immunoblotting image of BCL-2 and MCL1 in HL60 treated with Ctrl and AML-HS-5 CM for 0 h, 12 h, and 24 h. **I** Representative immunoblotting image of NFKB1, NFKB2, BCL2, and MCL1 in HL60 treated with Ctrl, AML-HS-5 CM, and shHDAC3 CM. **J** Representative immunoblotting image of NFKB1, NFKB2, BCL-2, and MCL1 in U937 treated with Ctrl, AML-HS-5 C,M, and shHDAC3 CM. All blots were normalized with a loading control. Data are presented as the mean ± SD. *n* = 3, *p < 0.05, **p < 0.01.

### HDAC3 inhibition delays cellular senescence of BMSCs and downregulates NFKB-MCL1 pathway in AML

Excessive accumulation of ROS in cells usually triggers continuously activation of NF-κB pathway and may eventually lead to the overexpression of inflammatory cytokines and cellular senescence [35–37]. We therefore examined the expression of genes associated with the NF-κB pathway of BMSCs treated with AML CM. As expected, a 3-day incubation of HS-5 cells with AML CM led to a notable rise in protein expression of NFKB1 (P105 and P50) and NFKB2 (P100 and P52). Inhibition of HDAC3 by BG45 down-regulated the expression of NFKB1 and NFKB2 protein induced by AML CM (Fig. 6A). Next, we examined the senescence of BMSCs after AML CM treatment. GSEA performed on senescence-related gene sets revealed HDAC3 inhibition suppressed oxidative stress induced senescence (NES = −1.17) and senescence associated secretory phenotype SASP (NES = −1.36) (Fig. 6B). β-Gal staining further showed that the induction of AML CM accelerated the senescence of primary BMSCs, which could be delayed by the addition of BG45 (Fig. 6C). When we used siRNA to interfere with RAB5A expression, we found that its NFKB2 expression level and phosphorylated iKBa (piKBa) level were reduced, indicating that RAB5A interference can inhibit the activation of the NFKB pathway (Fig. 6D). Notably, supplementation of RAB5A in shHDAC3 cells restored NF-κB pathway activation and promoted the secretion of IL6 and IL1B, further indicating that HDAC3 regulates NF-κB activation in HS-5 cells by modulating RAB5A levels (Fig. 6E and Fig. S6D).

Next, we tested whether the inhibition of HDAC3 in BMSCs could potentially deactivate the cytokine-driven survival signaling of AML. As shown in Fig. 6F, G, AML-BMSCs CM significantly promoted NF-KB2 activation of AML and the transcription of the downstream anti-apoptotic molecule MCL1. Further, AML-BMSCs CM promoted MCL1 protein accumulation in a time-dependent manner without significant effect on levels of BCL2 (Fig. 6H). shHDAC3 in HS-5 blocked the NFKB2 activation and reduced protein levels of the anti-apoptotic genes MCL1 and BCL2 in AML cell lines, including HL60 and U937 (Fig. 6I, J). As MCL1 as a key regulator of the compensation mechanism against apoptosis upon BCL2 inhibition [38], MCL1 inhibition induced by HDAC3 may be a key factor for synergistic killing of AML cells with venetoclax. Taken together, our results suggest that targeting HDAC3 can downregulate the release of inflammatory cytokines, thereby reversing the pro-AML survival of BMSCs.

### BMSCs-HDAC3 inhibition decreases AML proliferation and synergizes the anti-AML effect of venetoclax

**Fig. 7:**
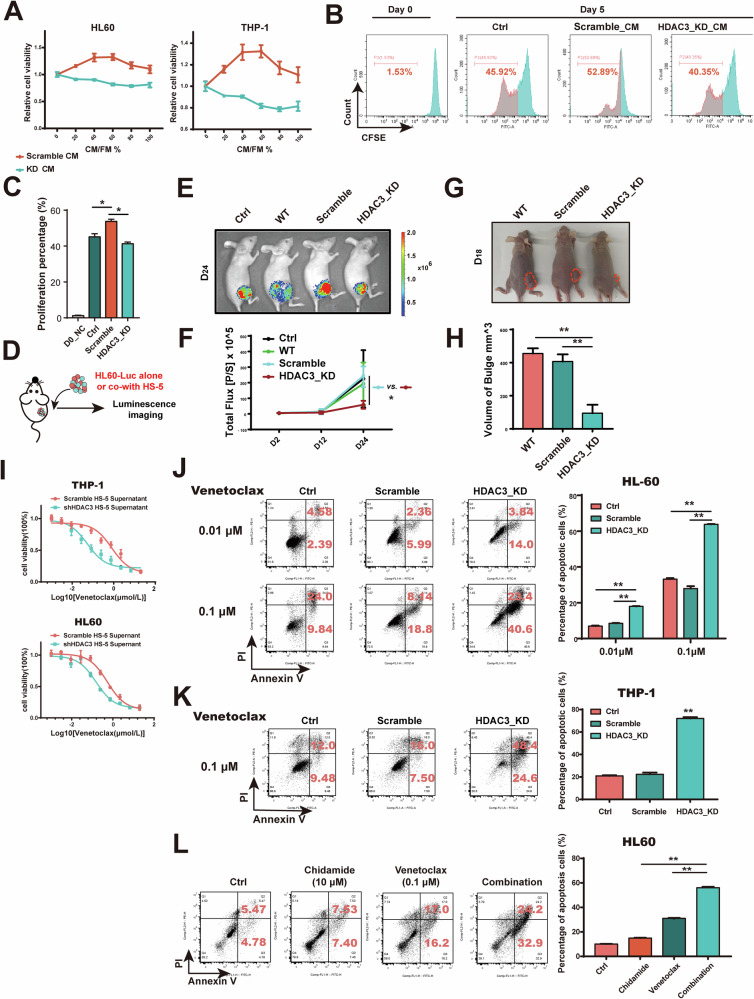
HDAC3 inhibition of BMSCs blocks the proliferation of AML and synergistically enhances the anti-AML effect of venetoclax. **A** Relative cell viability of AML cells (HL60 and THP-1) after treatment with different concentrations of shHDAC3 or scramble conditioned medium. **B**, **C** Flow cytometry results plot of CFSE-labeled HL60 treated with shHDAC3 or scramble conditioned medium. **D** Schematic diagram of animal modeling. **E** Bioluminescent representation of the tumor-bearing mice on day 24. **F** Tumor growth-time plot of tumor-bearing mice. **G** Representative image of a subcutaneous tumor in a mouse on day 18. **H** Statistical graph of subcutaneous tumor volume in tumor-bearing mice on day 18. **I** Dose-response curve of AML cells (THP-1 and HL60) treated by Venetoclax in the presence of HS-5 Scramble or shHDAC3 CM. **J**, **K** The cell apoptosis rate of HL60 and THP-1 induced by venetoclax in the presence or absence of HS-5 shHDAC3 CM. **L** CM from HS-5 was pretreated with Chidamide (10 μM) or not and then added to AML culture system together with Venetoclax or not, the cell apoptosis was detected by flow cytometry after 48 h. Data are presented as the mean ± SD. *n* = 3, *p < 0.05, **p < 0.01.

Given that HDAC3 inhibition in BMSCs reduced the production of inflammatory cytokines, we next investigated the effects of targeting HDAC3 in BMSCs on AML pathogenesis. We first showed that the use of shHDAC3 HS-5 conditoned medium led to decreased AML cell proliferation, with the most pronounced inhibitory effect observed at a 50% concentration of CM (Fig. 7A). Consistently, CFSE proliferation tracing experiments confirmed that shHDAC3 HS-5 CM inhibited the proliferation of HL60 cells (Fig. 7B, C). We further explored the impact of targeting HDAC3 in the tumor micro-environment using an AML-BMSC co-culture xenograft model. This model was established by co-injecting luciferase-expressing HL60 (HL60-Luc) cells and HS-5 cells into the right flank of Balb/c nude mice (Fig. 7D). The results indicated that HDAC3 depletion in BMSCs resulted in significant antitumor activity in vivo, leading to reduced AML growth over 24 days (Fig. 7E–H), supporting our in vitro findings that HDAC3 silencing in BMSCs inhibits AML cell proliferation. Recognizing the potential for higher therapeutic efficacy by targeting both cancer cells and the bone marrow niche, we examined whether targeting HDAC3 in BMSCs could enhance the anti-AML effects of venetoclax. We first assessed the dose-response of AML cells to venetoclax in the presence of scramble or shHDAC3 HS-5 CM. The results revealed that shHDAC3 HS-5 CM significantly lowered the half-maximal inhibitory concentration (IC_50_) of venetoclax in AML cells (Fig. 7I). Flow cytometry analysis further demonstrated that shHDAC3 HS-5 CM markedly enhanced venetoclax-induced apoptosis in both HL60 and THP-1 cells (Fig. 7J, K). Consistent with these findings, combining venetoclax with CM from HS-5 cells pretreated with chidamide (10 μM) led to a higher rate of apoptosis in HL60 cells compared to venetoclax alone (Fig. 7L). These findings collectively demonstrate that both genetic and pharmacological inhibition of HDAC3 in BMSCs suppresses AML proliferation and synergizes with venetoclax to exert a stronger anti-AML effect.

## Discussion and conclusion

AML is refractory to cure due to the continuous pro-survival and proliferative signaling from the bone marrow niche. Recently, novel combination treatments that target not only the cancer cells but also the BM-niche have shown an unexpected efficacy in AML [39]. Therefore, targeting the bone marrow micro-environment in conjunction with anti-AML therapy may result in better therapeutic outcomes. In this study, we propose that targeting HDAC3 in BMSCs is a promising approach to disrupt the AML-supportive niche and enhance anti-tumor therapy. Inhibiting HDAC3 expression can regulate oxidative stress damage caused by AML and reduce the excessive release of inflammatory cytokines through delaying cellular senescence, thereby weakening the tumor-supporting effect of BMSCs.

The comparison between sequencing results of shHDAC3 HS-5 cells and the GEO database further demonstrated that RAB5A may be a key molecule regulated by HDAC3. RAB5A is a small GTPase involved in early endosome formation [40] and has been shown to promote drug resistance in gastric cancer by inhibiting autophagy [41]. It also plays a role in autophagolysosome fusion, where its constitutively active form can inhibit autophagosome-lysosome fusion, thereby blocking autophagic flux [42]. In addition, suppression of RAB5A expression has been reported to restore saikosaponin D (SsD)-induced autophagosome lysosome fusion block, which further points out the key role of RAB5A in autophagy regulation [43]. Mitochondrial autophagy (mitophagy) is a type of autophagy to maintain mitochondrial homeostasis. Beyond its role in general autophagy, no studies have clarified the role of RAB5A in mitophagy. AMPK is a monitoring element that regulates mitochondrial metabolism and homeostasis [44]. Activation of AMPK contributes to the activation of mitochondrial autophagy [45]. In our study, RAB5A inhibition led to increased expression of p-AMPK and total AMPK, along with decreased levels of p62 and LC3B, indicating enhanced mitophagy. Notably, re-expression of RAB5A restored PINK1 levels and increased TOMM20 accumulation, suggesting that RAB5A functions to suppress mitochondrial autophagy. Our work also directly reveals that RAB5A is a downstream factor of YY1. HDAC3 stabilizes YY1 by catalyzing acetylation and ubiquitination of YY1, therefore enhancing the transcriptional activity of YY1 to induce RAB5A expression. Hence, our study identified HDAC3-RAB5A signaling as a novel regulating mechanism in the regulation of mitochondrial autophagy.

Our study also provides new insights into the relationship between AML-BMSC mitochondrial autophagy and inflammation. The mitochondrial autophagy pathway can remove the damaged mitochondria and reduce the damage to cells. Promoting the process of mitochondrial autophagy can effectively reduce the inflammatory response [46, 47]. Studies on BMSCs have pointed out that by promoting mitochondrial autophagy of BMSCs cells, it helps to improve mitochondrial function and inhibit BMSCs aging [48]. During the aging process of cells, the persistent activation of pathways, including NF-κB signaling pathway [49], leads to the upregulation of anti-apoptotic proteins and impede the removal of senescent cells, thereby inducing intractable inflammation due to the release of a substantial amount of SASP from senescent cells. Our analysis of the GSE107490 dataset, along with subsequent RNA sequencing on shHDAC3 HS-5 cells, also revealed enrichment of gene sets related to inflammation, pointing to a severe inflammatory response in BMSCs during AML-induced cell aging. Additionally, our results demonstrate that the addition of the HDAC3 inhibitor BG45 can alleviate inflammation by suppressing the activation of NF-κB pathways and reducing the number of senescent cells. These findings provide valuable insight into the mechanisms of AML-induced cell aging in BMSCs and suggest that inhibiting HDAC3 may be a promising strategy for mitigating cellular senescence induced by ROS.

Improving the tumor micro-environment is a combination anti-tumor therapy with 1 + 1 > 2 therapeutic potential. In a clinical study on venetoclax and azacitidine resistance in AML, it was found that chidamide can enhance the therapeutic effects of venetoclax and azacitidine without causing significant side effects, which may be attributed to chidamide-mediated downregulation of MCL1 levels in AML cells [50]. In our study, we further demonstrate that inhibiting HDAC3 expression in BMSCs mitigates AML-induced mitochondrial oxidative stress by promoting mitochondrial autophagy, thereby restoring BMSC function. Additionally, HDAC3 inhibition in BMSCs reduces the AML-induced upregulation of MCL1 in AML cells, thereby enhancing the therapeutic efficacy of venetoclax. Thus BMSC-HDAC3 and its regulatory pathways is a potential new therapeutic avenue for targeting the bone marrow micro-environment in conjunction with anti-AML therapy. Inhibition of HDAC3 has already been shown to have significant anti-leukemia effects. Targeted inhibition of HDAC3 can counteract drug resistance in acute promyelocytic leukemia (APL) by promoting the degradation of PML-RARα [51]. Ferroptosis of stem cell-like leukemia cells in AML can be induced by combining HDAC3 inhibitors with PPAR agonists [52]. Our study provides new insights into dual effects of HDAC3 inhibitors of remodeling the bone marrow micro-environment and inhibiting tumor progression, which provides a new theoretical basis for the subsequent clinical use of combined with other drugs.

In conclusion, our findings provide new insights into the role of HDAC3 in regulating mitochondrial homeostasis and inflammatory responses in BMSCs and its potential impact on AML cells, which may open up new avenues for the development of AML therapy.

## Materials and methods

### Cell lines and cell culture

All primary material including peripheral blood and bone marrow extract was collected following informed consent and strict adherence to the ethical principles outlined in the Helsinki Declaration of the World Medical Association. Studies using patient samples were approved by the ethical advisory board of Medical Ethics Committee of Zhujiang Hospital of Southern Medical University (No. 2022-KY-220-01).

Human primary AML cells were obtained as previously described [53]. HS-5, C1498, HL60, KG1A, THP-1 and U937 cells lines were purchased from Cellcook. HL60, U937, THP-1 and KG1A were cultured with RPMI1640 (Invitrogen) containing 10% FBS (Gibco), 100 units/mL penicillin, and 100 µg/mL streptomycin (Gibco). HS-5 cells were cultured with DMEM (Invitrogen) containing 10% FBS (Gibco), 100 units/mL penicillin, 100 µg/mL streptomycin (Gibco), and 1×non-essential amino acid (Solarbio). C1498 cells were cultured with DMEM (Invitrogen) containing 10% FBS (Gibco), 100 units/mL penicillin and 100 µg/mL streptomycin (Gibco). All cells are STR verified. AML-BMSCs were induced by culturing HS-5 stromal cells with AML cells derived conditioned medium for 3 days. All cells were cultured in a humidified incubator at 37 °C with 5% CO_2_.

### Lentiviral shRNA production and infection

Information for the shHDAC3 lentiviral vector is shown in Supplementary Table S1. To generate the shRNA lentivirus targeting HDAC3, we first co-transfected recombinant packaging plasmids with shHDAC3 plasmids into HEK293T cells. We collected culture supernatants containing the virus 48 and 72 h after transfection. To infect HS-5 cells with the lentivirus, we cultured the cells in the presence of the lentivirus solution with a final concentration of 8 μg/mL of polybrene (Sigma) for 24 h. Finally, cells stably expressing target plasmids were screened by puromycin at 3 mg/mL.

### Western blotting

Cells were lysed with RIPA lysis buffer (Beyotime) containing protease inhibitor (Roche) and phosphatase inhibitor (Beyotime). And then the protein concentration was measured by Bradford protein assay. Protein was separated using sodium dodecyl sulfate–polyacrylamide gel electrophoresis (SDS–PAGE) and transferred to PVDF membrane (Millipore). The membranes were sealed with 5% nonfat milk at room temperature for 2 h and incubated overnight with the corresponding primary antibodies. HRP-conjugated anti-rabbit or anti-mouse secondary antibodies was added and incubated for 1 h at room temperature. Finally, the blots were detected using an ECL Chemiluminescent Substrate Reagent kit (Protein Tech) and analyzed with ImageJ software. The information of antibodies can be found in Table S2 in supplemental materials.

### RNA isolation and qPCR analysis

Total RNA was extracted from the cells using TRIzol (Vazyme) according to the manufacturer’s instructions. The RNA concentration and purity were measured with a NanoDrop One trace spectrophotometer (Thermo Fisher Scientific). RNA was reversely transcribed into cDNA and then quantified by qPCR using 2 × SYBR Green qPCR Mix (Trans Gen Biotech) with Light Cycler 96 (Roche). Finally, fold changes of relative gene expression were analyzed by using the 2 −^ΔΔCt^ method. The qPCR primers used in this experiment are given in Supplemental Table S3.

### Cell viability, proliferation, and apoptosis Assays

Carboxyfluorescein succinimidyl amino ester (CFSE) staining, cell counting kit-8 (CCK-8) and apoptosis detection kit were applied to analyze cells viability.

Cell proliferation was assessed by tracking the fluorescence intensity of CFSE-stained AML cells using flow cytometry, as previously described [54]. Briefly, AML cells were labeled with CFSE (5 μM) and then treated with either control medium or AML-HS conditioned medium. After three days or a specified time interval, the cells were harvested and analyzed by flow cytometry to determine the fluorescence intensity of the CFSE-labeled cells. The results obtained from the flow cytometry analysis were used to calculate the rate of cell proliferation.

Apoptotic cells were detected using AnnexinV/propidium iodide (PI) kit (BD Biosciences) according to operation guide. Briefly, AML cells were harvested after being treated with venetoclax in the presence or absence of shHDAC3 conditioned medium for 24 h. Then, AML cells were incubated with Annexin V-FITC and PI for 15 minutes and immediately subjected to flow cytometry analysis to determine the percentage of apoptotic cells.

The CCK-8 assay was used to determine the cell viability of AML cells treated with control medium, AML-HS CM, or shHDAC3-HS CM. Briefly, AML cells were seeded into a 96-well plate and treated with the respective conditioned media. After 48 h of treatment, the plate was removed from the incubator, and 10 μL of the CCK-8 reagent was added to each well. The plate was then incubated for an additional 2-4 h and subjected to the absorbance measurement at 450 nm using a micro-plate reader.

### Mitochondrial function detection

JC-1 staining: JC-1 was purchased from Solarbio Biotech. Cells were seeded into 6-well plates and treated according to experimental groups. After treatment, cells were collected and washed twice with PBS. According to the instructions of the JC-1 kit, JC-1 staining working solution (5 μM) was added to the cell suspension and incubated at 37 °C for 20-30 minutes, avoiding light. After incubation, the staining solution was discarded and the cells were washed twice with PBS. Fluorescence signals were detected by flow cytometry. When the mitochondrial membrane potential is high, JC-1 exists in aggregates and emits red fluorescence (Ex/Em = 585/590 nm); when the mitochondrial membrane potential decreases, JC-1 exists in monomers and emits green fluorescence (Ex/Em = 510/527 nm). Therefore, the red/green fluorescence ratio can be used to evaluate the functional status of mitochondria.

Mitochondrial superoxide detection: HKSOX-1m was purchased from MCE (USA). Cells were seeded into 6-well plates and treated according to experimental groups. After treatment, cells were collected and washed twice with PBS. Similarly, HKSOX-1m staining working solution was added to the cell suspension and incubated at 37 °C for 20 minutes, avoiding light. After incubation, the staining solution was discarded and the cells were washed twice with PBS. The fluorescence signal was detected by flow cytometry.

### Transcriptome sequencing

Transcriptome sequencing was entrusted to Gene Denovo Biotechnology Co. (Guangzhou, China) for the services including RNA quantification and qualification, library construction, sequencing, and raw data analysis. Briefly, raw data (in fastq format) were firstly subjected to primary quality control to obtain clean data (clean reads). For read quality control, fastp was used to process the raw reads by filtering out low-quality data to obtain clean reads [55]. The filtering steps included removing adapter-containing reads, discarding reads with an N ratio greater than 10%, eliminating poly-A reads, and excluding low-quality reads in which more than 50% of bases had a quality score of Q ≤ 20. The clean, paired-end reads were then aligned to the reference genome using TopHat as the mapping tool. TopHat generates a database of splice junctions based on the gene model annotation file, leading to better mapping results compared to other non-splice mapping tools. Finally, HTSeq (version = 2.0.5) was employed to count the reads mapped to each gene.

### Gene set enrichment analysis (GSEA)

Gene expression matrixes retrieved from GEO database or obtained from RNA sequencing were loaded into the GSEA software (version 4.1.0) with group information, the data was then analyzed to identify patterns and relationships between gene expression and various biological processes based on gene sets from molecular signature database.

### KEGG enrichment analysis

Differentially expressed genes (DEGs) based on our RNA-seq or downloaded data from GEO were calculated by DeSeq2 (version = 1.38.3) or limma (version = 3.54.2) R package. And then clusterProfiler (version = 4.6.0) was utilized to map the DEG list to KEGG pathway IDs. The Benjamini-Hochberg (BH) method was used to correct multiple testing and control the false discovery rate. Finally, the results were visualized using a scatterplot or bar chart showing the enriched pathways ranked by significance level.

### Mice study

All protocols for mouse experiments were approved by the animal ethics committee of Sun Yat-Sen University, China. The approved number was SYSU-IACUC-2021-000701. Briefly, Balb/c nude mice were randomly divided into four groups: AML cells transplantation alone, AML co-transplanted with WT-HS-5 cells, AML co-transplanted with scramble-HS-5 cells, and AML co-transplanted with shHDAC3-HS-5 cells. AML cells (2 × 10^6^) alone or in combination with HS-5 cells (5×10^5^) were mixed with Matrigel (Corning, Cat. No. 354248) at a ratio of 1:1 and subcutaneously injected into the flank of the nude mice. Bioluminescence imaging was performed on day 24 after transplantation.

### Statistical analysis

The data were presented as mean ± standard deviation (SD) of at least three independent experiments. Statistical analysis was performed using one-way analysis of variance (ANOVA) for normally distributed data. When normality could not be confirmed or the sample size was small, non-parametric tests, including the Mann-Whitney U test or the Kruskal-Wallis test with post-hoc analysis, were applied. Additionally, Tukey’s test and unpaired Student’s *t*-test were used for multiple and pairwise comparisons, respectively.

### Data statement

The raw data of RNA-seq result can be found in China National Center for Bioinformation and the data retrieval number is HRA005414. Other data that support the findings of this study are available from the corresponding author upon reasonable request.

## Supplementary information

Supplemental data

WB metadata
