# Supplementary material for: HDAC3-YY1-RAB5A axis remodels AML-supportive niche by modulating mitochondrial homeostasis in bone marrow stromal cells
Source: Cell Death Dis. 2025 Jul 7;16(1):498. doi: 10.1038/s41419-025-07777-9 (PMC12234896; doi:10.1038/s41419-025-07777-9)
Supplement: Supplementary file 1 — Supplemental data [file 41419_2025_7777_MOESM1_ESM.docx]

**Supplemental data**

**HDAC3-YY1-RAB5A axis remodels AML-supportive niche by modulating mitochondrial homeostasis in bone marrow stromal cells**

Chao He^1,2┲^, Yue Xiong^1, 3┲^,Yuqing Zeng^1^, Jianhua Feng ^4, 5^, Fuxia Yan^1^, Manqi Zhang^1^, Zhili Tan^4^, Yaling Zheng^4^, Hongbo Chen^1, *^, Rui Huang^4, *^, Fang Cheng^1, *^

1. School of Pharmaceutical Sciences (Shenzhen), Sun Yat-sen University, Shenzhen, 518107, P.R. China.
2. Institute of Chemical Biology, Shenzhen Bay Laboratory, Shenzhen 518107, China.
3. The Eighth Affiliated Hospital, Sun Yat-sen University, 3025 Shennan Middle Road, Shenzhen, 518000, China.
4. Department of Hematology, Zhujiang Hospital of Southern Medical University, Guangzhou, 510282, P.R. China.
5. The Affiliated Dongguan Songshan Lake Central Hospital, Guangdong Medical University, Dongguan, Guangdong, 523326, China.

*Correspondence to: [chengf9@mail.sysu.edu.cn](mailto:chengf9@mail.sysu.edu.cn) (F. Cheng) #

[rachelchn@163.com](mailto:rachelchn@163.com) (R. Huang)

[chenhb7@mail.sysu.edu.cn](mailto:chenhb7@mail.sysu.edu.cn) (H. Chen)

#will handle correspondence at all stages of refereeing and publication, also post-publication.

<Tel:+86-15527709102>;

Competing Interests statement

The authors declare no competing financial interest.

^┲^ Contributed equally.

### Methods and Materials

### Methods

**1.1.1 ChIP-PCR**

DNA pull down kit (Fitgene, FI8903) was used to detect the binding of YY1 and RAB5A promoter. Briefly, cells (2 × 10^7^) were collected and cross-linked with 1% formaldehyde to preserve protein-DNA interactions. The reaction is then quenched with glycine, and the cells are lysed to extract chromatin. Next, chromatin is sheared on ice *via* sonication using a probe sonicator at 35% power in a cycle of 2 s sonication followed by a 5 s pause, repeated for a total of 15 minutes, ensuring fragment sizes between 200–1000 bp, which are confirmed by agarose gel electrophoresis. The lysate is then pre-cleared with Protein A/G magnetic beads before immunoprecipitation. For immunoprecipitation, chromatin is incubated overnight with a target-specific antibody, using IgG as a control. Immune complexes are captured with Protein A/G beads, followed by sequential washes with low-salt and high-salt buffers to remove non-specific binding. The DNA-protein complexes are then eluted, and cross-links are reversed by heating. DNA is purified using 10 × TE buffer, a phenol : chloroform : isoamyl alcohol (25 : 24 : 1) mixture (400 µL), NaCl ethanol solution, and 80% ethanol precipitation. The purified DNA pellet is finally dissolved in 20 µL ddH_2_O. For analysis, qPCR is performed using the ^ΔΔ^Ct method to quantify enrichment levels.

**1.1.2 Detection of IL6 and IL1B**

The ELISA kits for IL-6 (S0C3004) and IL-1B (S0C3013) were purchased from STARTER. HS-5 cells were seeded in 6-well plates and incubated for 24 h. The cell supernatant was then harvested and centrifuged at 2000g for 10 minutes to remove cells and debris. The clarified supernatant was transferred to a new centrifuge tube for further analysis. Following the manufacturer's instructions, 50 µL of each sample (diluted 1:200 for IL-6 and 1:10 for IL-1B) was mixed with 50 µL of antibody solution and added to a pre-coated 96-well plate. The plate was incubated at 37°C for 45 minutes, followed by 3 – 5 washes. After washing, TMB substrate was added, and the plate was incubated at 37°C for 5 – 10 minutes before adding the stop solution. The absorbance was measured at 450 nm, and cytokine concentrations were determined using a standard curve.

### Materials

### **1.**2.1 Information of shRNA vectors

### TableS1. Information of shRNA vectors

| **Name** | **Sequence** |
| --- | --- |
| ShHDAC3-1 | ***ACCGGGCACCATGCCAAGAAGTTTGACTCGAGTCAAACTTCTTGG CATGGTGCTTTTTTGAATTC*** |
| ShHDAC3-2 | ***ACCGGCCTTCCACAAATACGGAAATTCTCGAGAATTTCCGTATTTG TGGAAGGTTTTTTGAATTC*** |
| ShHDAC3-3 | ***ACCGGGGAGCTTCCCTATAGTGAATACTCGAGTATTCACTATAGG GAAGCTCCTTTTTTGAATTC*** |
| shNC | ***ACCGGTTCTCCGAACGTGTCACGTTTCAAGAGAACGTGACACGTTCG GAGAATTTTTTGAATTC*** |

Name of vetcor: pLKD-U6-MCS CMV-EGFP2A-Puro

### **1.**2.2 Antibodies

TableS2. List of antibodies

| Name | Manufacturer | Catalog No. |
| --- | --- | --- |
| *Anti-HDAC3* | ***Abways*** | ***CY5595*** |
| *Anti-GAPDH* | ***Zen Bioscience*** | ***200306-7E4*** |
| *Anti-NFKB1* | ***CST*** | ***13586*** |
| *Anti-NFKB2* | ***CST*** | ***4882*** |
| *Anti-BCL2* | ***Affinity*** | ***BF9103*** |
| *Anti-MCL1* | ***CST*** | ***94296*** |
| *Anti-RAB5A* | ***CST*** | ***46449*** |
| *Anti-YY1* | ***CST*** | ***D5D9Z*** |
| *Anti-LC3B* | ***CST*** | ***2775*** |
| *Anti-P62* | ***Abmart*** | ***T55546S*** |
| *Anti-AMPK* | ***Abmart*** | **TU405340S** |
| *Anti-p-AMPK* | ***Abmart*** | **TA3423S** |
| *Anti-ACTIN* | ***ABclonal*** | ***AC004*** |
| *Acetylated-Lysine Mab* | ***Abmart*** | ***M30069S*** |
| *Anti-Pan-ubiquitin* | ***Proteintech*** | ***10201-2-AP*** |
| *Anti-p-IKBa* | ***Zen Bioscience*** | ***370348*** |
| *Anti-Flag Tag Agarose conjugated* | ***Abmart*** | ***M20038S*** |
| *Anti-PINK-1* | ***Selleck*** | ***F0490*** |
| *Anti-TOMM20* | ***Selleck*** | ***F0513*** |

### **1.**2.3 qPCR analysis

TableS3. List of primers

| Gene | Forward primer | Reverse primer |
| --- | --- | --- |
| *human-ACTB* | ***CATGTACGTTGCTATCCAGGC*** | ***CTCCTTAATGTCACGCACGAT*** |
| *human-GAPDH* | ***GGAGCGAGATCCCTCCAAAAT*** | ***GGCTGTTGTCATACTTCTCATGG*** |
| *human-HDAC1* | ***TGGAAATCTATCGCCCTCAC*** | ***TCTCTGCATCTGCTTGCTGT*** |
| *human-HDAC2* | ***CTGTTAATTGGGCTGGAGGA*** | ***AATTCAAGGATGGCAAGCAC*** |
| *human-HDAC3* | ***GAGGGATGAACGGGTAGACA*** | ***CAGGTGTTAGGGAGCCAGAG*** |
| *human-HDAC10* | ***AGAAACACGGGCTACACAGG*** | ***CGGTGCCAGGAGAAGTAAAG*** |
| *human-IL6* | ***ACTCACCTCTTCAGAACGAATTG*** | ***CCATCTTTGGAAGGTTCAGGTTG*** |
| *human-IL8* | ***ACTGAGAGTGATTGAGAGTGGAC*** | ***AACCCTCTGCACCCAGTTTTC*** |
| *human-CCL2* | ***CAGCCAGATGCAATCAATGCC*** | ***TGGAATCCTGAACCCACTTCT*** |
| *human-IL1B* | ***ATGATGGCTTATTACAGTGGCAA*** | ***GTCGGAGATTCGTAGCTGGA*** |
| *human-BCL2* | **GGTGGGGTCATGTGTGTGG** | **CGGTTCAGGTACTCAGTCATCC** |
| *human-MCL1* | **TGCTTCGGAAACTGGACATCA** | **TAGCCACAAAGGCACCAAAAG** |
| *human-BCL-XL* | **GAGCTGGTGGTTGACTTTCTC** | **TCCATCTCCGATTCAGTCCCT** |
| *human-RAB5A* | ***CAAGGCCGACCTAGCAAATAA*** | ***GATGTTTTAGCGGATGTCTCCAT*** |
| *RAB5A-CHIP* | ***AAAGGAATTTCCCCCGACCC*** | ***CCTCTCCAGCGCCATTGTTA*** |

### Figures


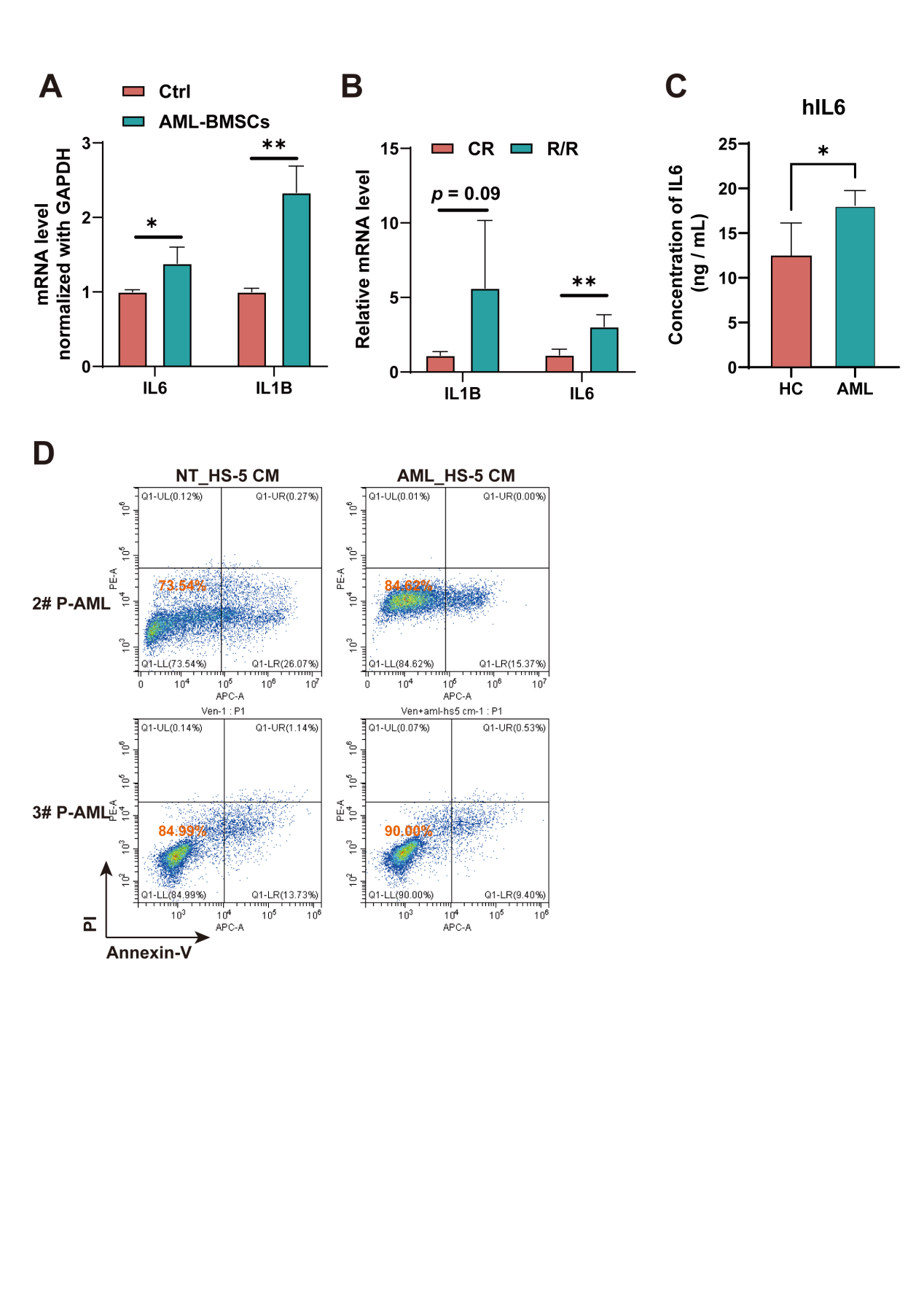


**FigS1. Inflammatory cytokines level of AML-BMSCs and its protective effect on AML cells. A-B)** The level of IL6 and IL1B in induced AML-BMSCs and BMSCs from CR/RR AML patients. **C)** The level of IL6 in serum from health Ctrl (HC) and AML patients. **D)** Apoptosis test of primary AML cells induced by Venetoclax (0.1 μM) in different treatment. Data are presented as the mean ± SD. *n* = 3, **p* < 0.05, ***p* < 0.01


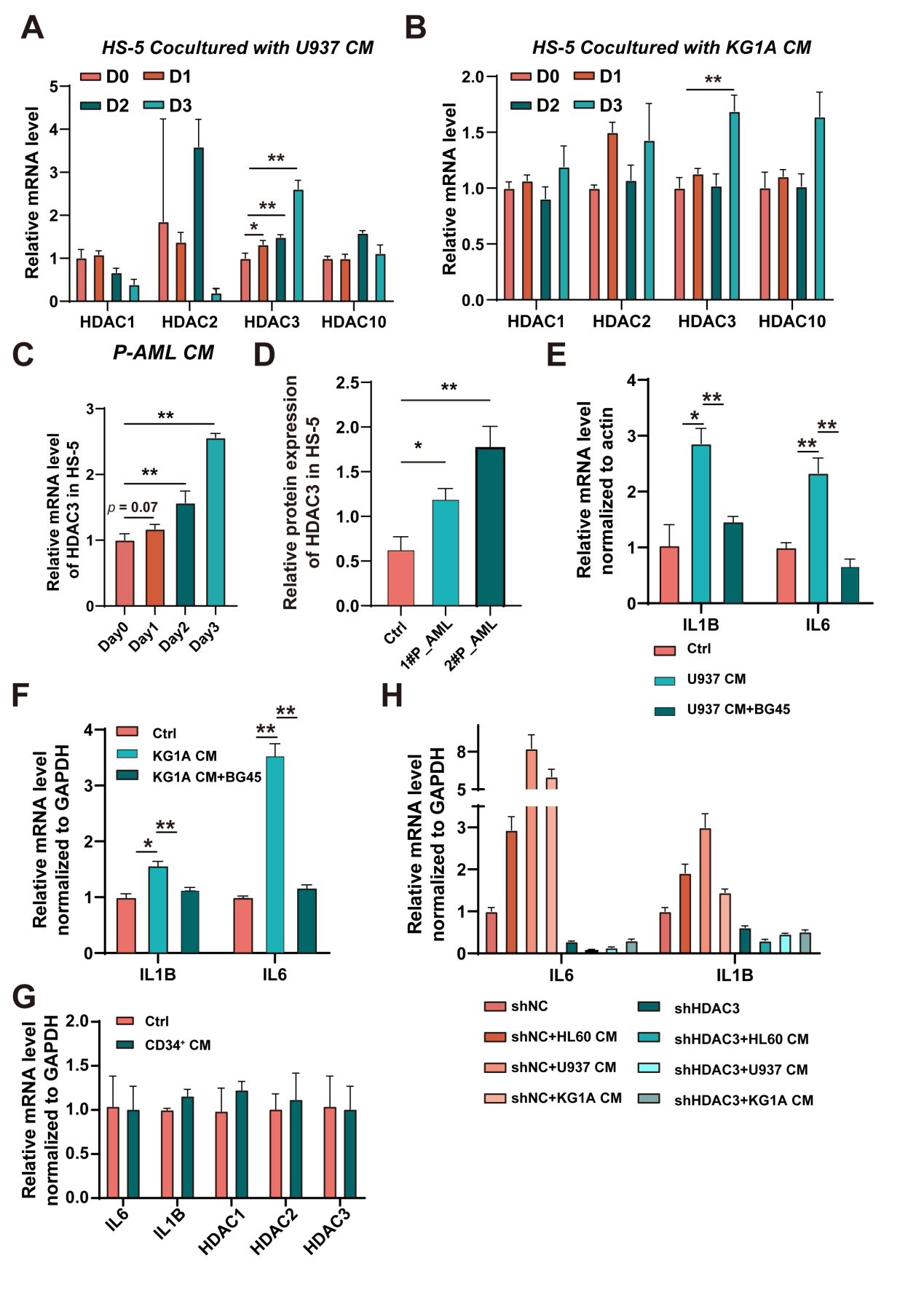


**FigS2. HDAC3 in BMSCs modulate the hyperinflammatory phenotype. A-B)** mRNA expression of HDACs in HS-5 cocultured with U937 and KG1A CM, respectively. **C)** mRNA expression of HDACs in HS-5 cocultured with AML patient cells derived CM. **D)** Protein expression of HDAC3 in HS-5 after being cultured with AML cell lines or AML patient cellsderived conditioned medium. The data was normalized to loading control. **E-F)** mRNA level of IL6 and IL1B in HS-5 induced by CM and CM+BG45. **G)** mRNA level of IL6, IL1B and HDACs in HS-5 cells induced by CD34+ cells derived CM. **H)** mRNA level of IL6 and IL1B in shNC and shHDAC3 HS-5 cells induced by AML cells derived CM and CM+BG45. Data are presented as the mean ± SD. *n* = 3, **p* < 0.05, ***p* < 0.01

**
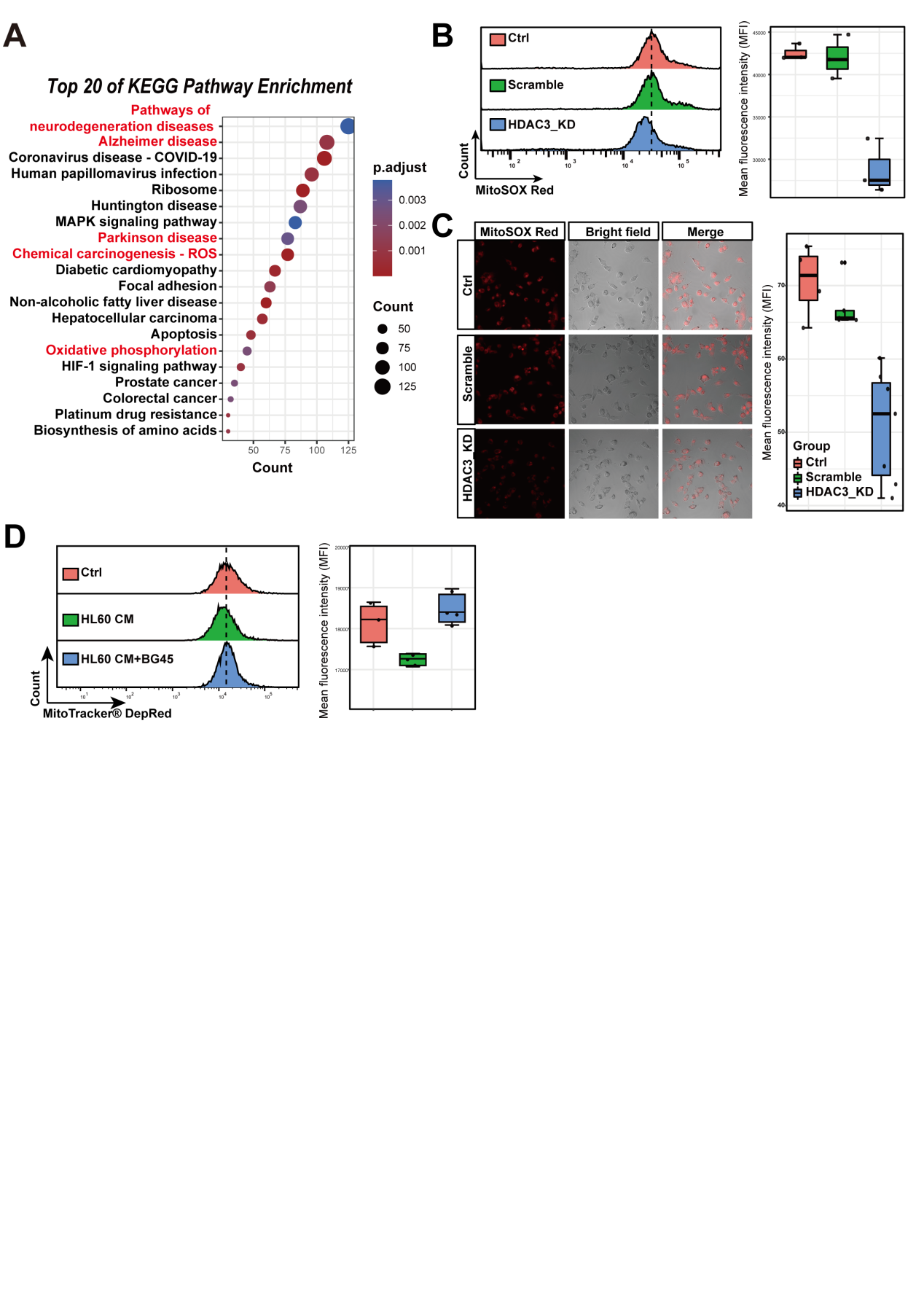
**

**FigS3. HDAC3 plays a crucial role in regulating the homeostasis of mitochondrial oxidative stress in BMSCs. (A)** KEGG enrichment analysis of genes downregulated in shHDAC3 group. (B) Flow cytometry analysis of mitROS level in HS-5 treated by ctrl, HL60 CM and HL60 CM+BG45 after 72 h. The right panel is the statistics of mean fluorescence intensity.(C) Representative confocal images of mitROS level in Ctrl, Scramble and shHDAC3 HS-5 cells. (D) Flow cytometry analysis of mitochondrial quality of HS-5 treated by ctrl, HL60 CM and HL60 CM+BG45 after 72 h. The right panel is the statistics of mean fluorescence intensity.


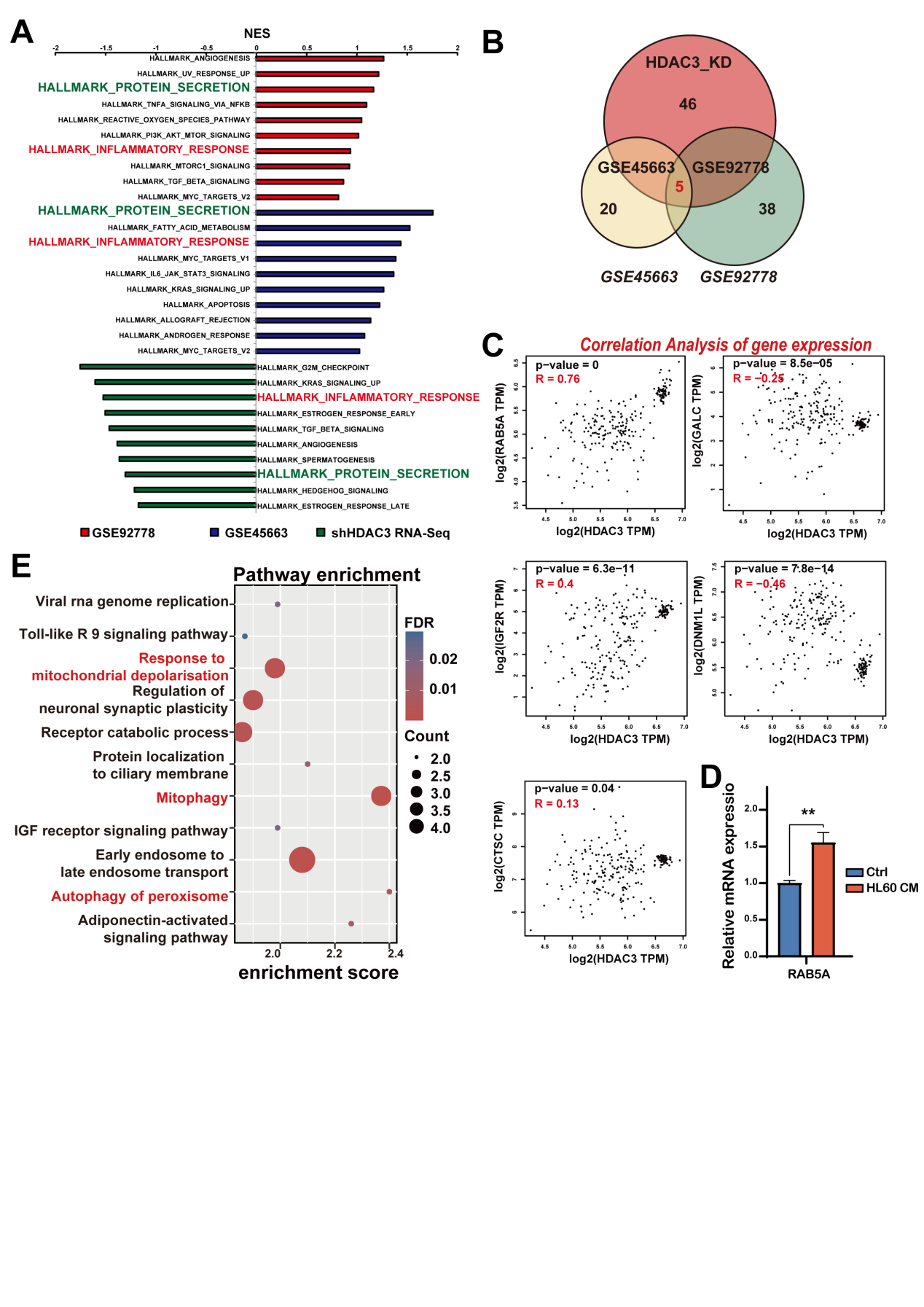


**FigS4. HDAC3 regulated sEVs secretion via RAB5A. (A)**The enriched terms of Hallmark genes set in GSE92778, GSE45663 and shHDAC3 RNA-Seq using GSEA. **(B)** The Venn diagram of genes significantly altered in the protein secretion-related gene sets in shHDAC3, GSE45663, and GSE92778 datasets. **(C)** Gene correlation analysis of HDAC3 and 5 co-enriched genes, RBA5A, IGF2R, DNM1L, GALC and CTSC using Gepia2 database (<http://gepia2.cancer-pku.cn/>). **(D)** Relative mRNA expression of RAB5A in HS-5 treated with Ctrl and HL60 CM. **(E)** KEGG pathway enrichment analysis of proteins interacting with RAB5A. Data are presented as the mean ± SD. *n* = 3, **p* < 0.05, ***p* < 0.01


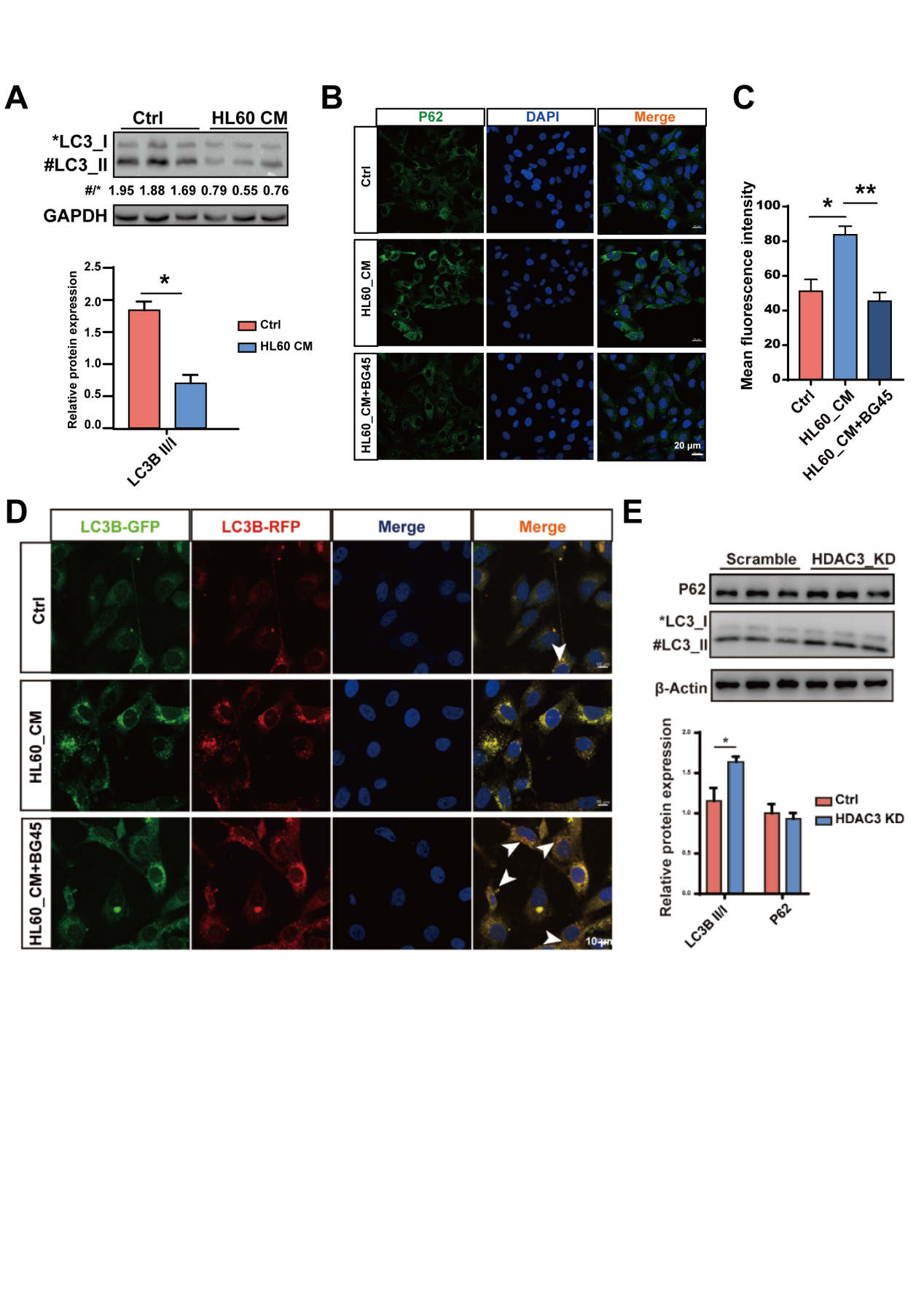


**FigS5. HDAC3 inhibition promoted autophagosome-lysosomal fusion in BMSCs treated with AML CM.** **(A)** Protein expression of LC3B in HS-5 treated with HL60 CM. **(B-C)** Representative confocal image of P62 in HS-5 treated with ctrl, HL60 CM, and HL60 CM+BG45; the right panel is the statistical image. Scale bar = 20 μm. **(D)** Representative confocal image of LC3B-GFP-RFP in HS-5 treated with Ctrl, HL60 CM, and HL60 CM+BG45. Scale bar =10 μm **(E)** Protein expression of LC3B and P62 in Scramble and shHDAC3 HS-5. Data are presented as the mean ± SD. *n* = 3, **p* < 0.05, ***p* < 0.01


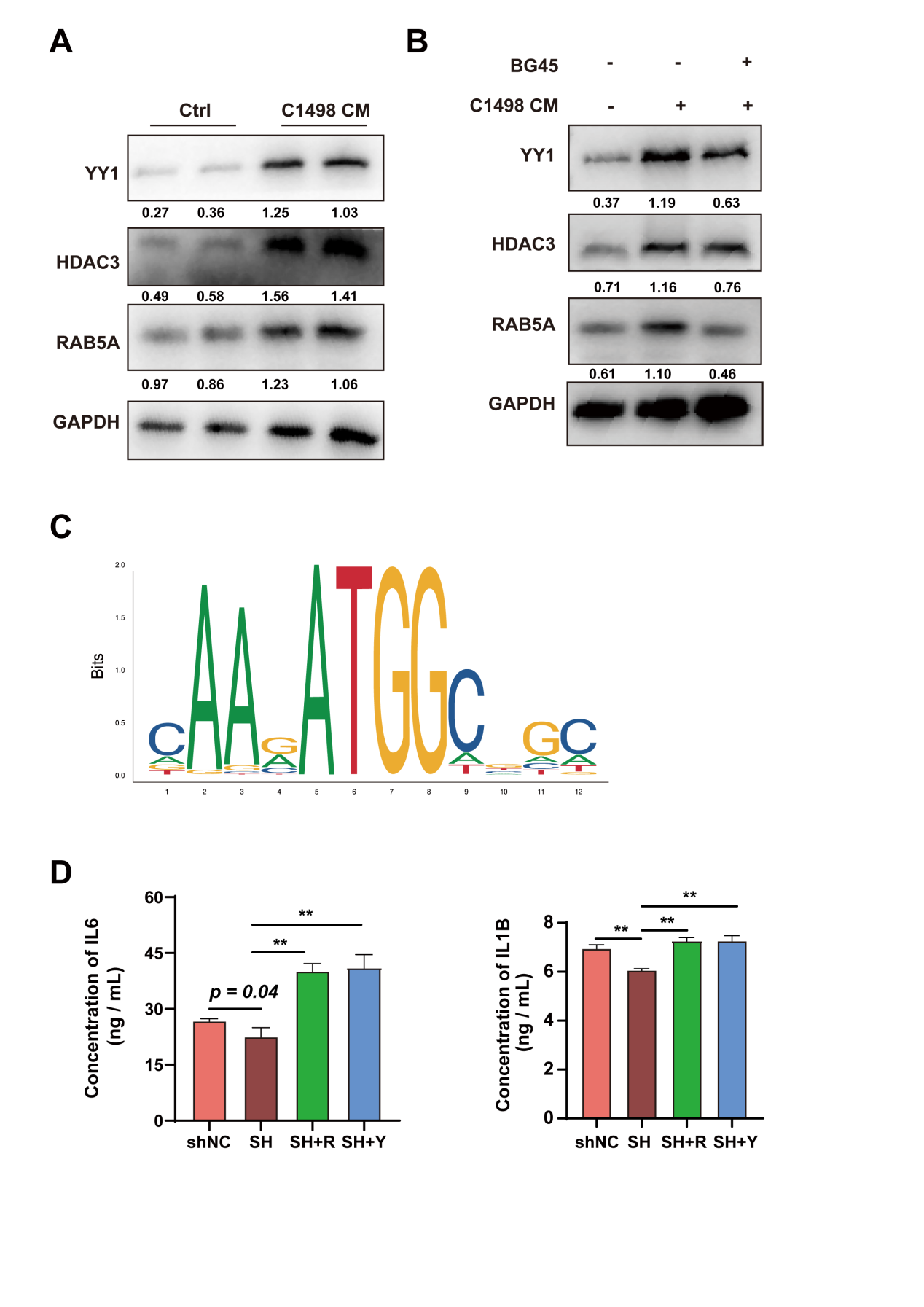


**FigS6. HDAC3 inhibits YY1 acetylation to modulate RAB5A.** **(A)** Immunoblots of YY1 and RAB5A in OP9 cells after treated by C1498 cells derived CM. **(B)** Immunoblots of YY1, HDAC3 and RAB5A in OP9 cells after treated by C1498 CM and CM+BG45. **(C)** Binding site of YY1 to the RAB5A promoter region as predicted by the JASPR database. **(D)** Concentration of IL6 and IL1B in CM derived from shNC, shHDAC3, shHDAC3+RAB5A and shHDAC3+YY1 HS-5 cells. Data are presented as the mean ± SD. *n* = 3, **p* < 0.05, ***p* < 0.01.
