# Supplementary material for: HDAC3-YY1-RAB5A axis remodels AML-supportive niche by modulating mitochondrial homeostasis in bone marrow stromal cells
Source: Cell Death Dis. 2025 Jul 7;16(1):498. doi: 10.1038/s41419-025-07777-9 (PMC12234896; doi:10.1038/s41419-025-07777-9)
Supplement: Supplementary file 2 — WB metadata [file 41419_2025_7777_MOESM2_ESM.pdf]

Fig2-D

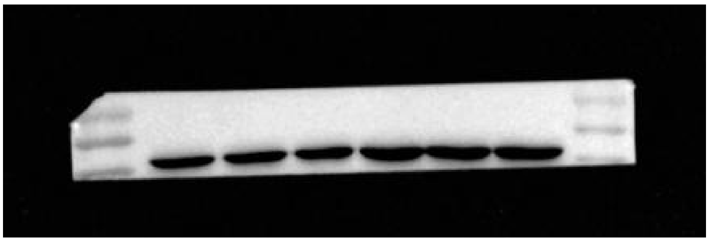

HL60-GAPDH

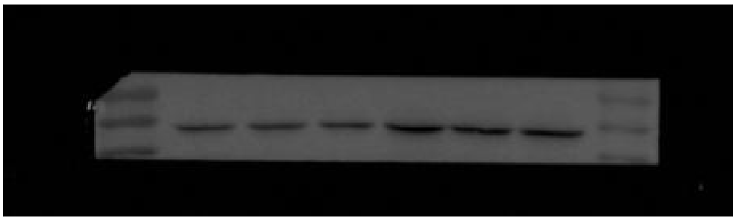

HL60-HDAC3

Fig2-F

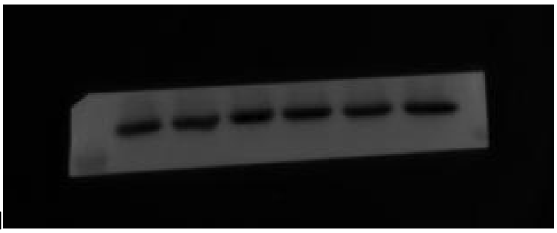

KG1A-GAPDH

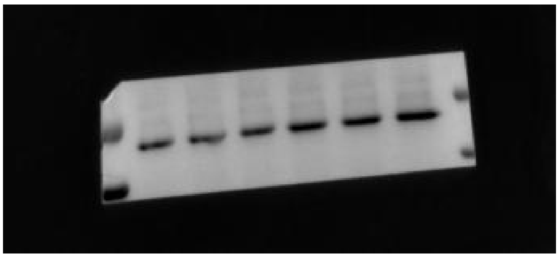

KG1A-HDAC3

Fig2-E

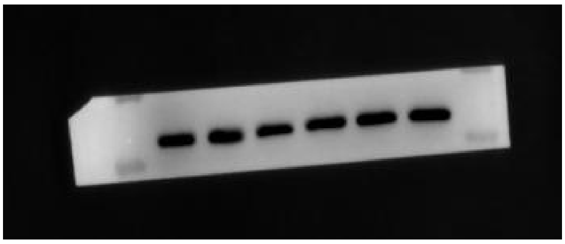

U937-GAPDH

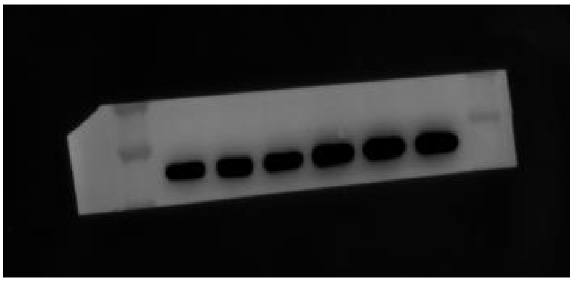

U937-HDAC3

Fig2-G

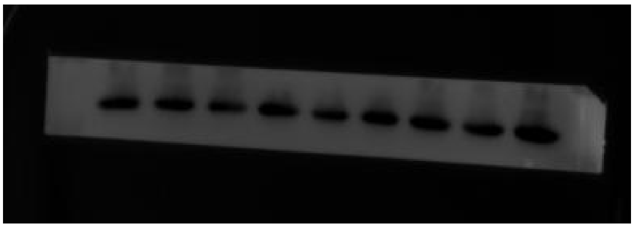

GAPDH

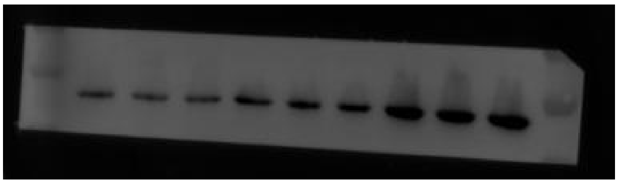

HDAC3

Fig2-H

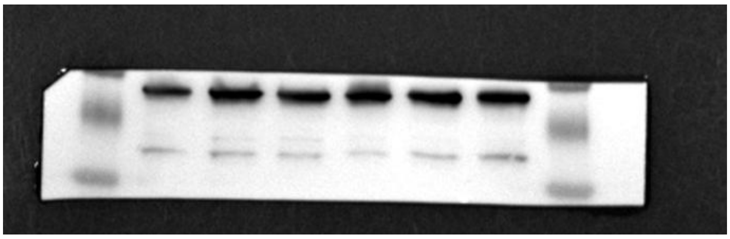

GAPDH

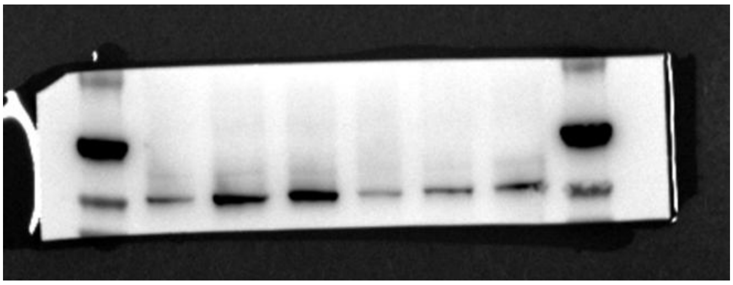

HDAC3

Fig4-C

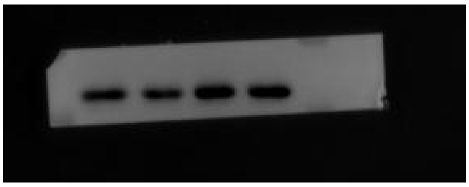

GAPDH

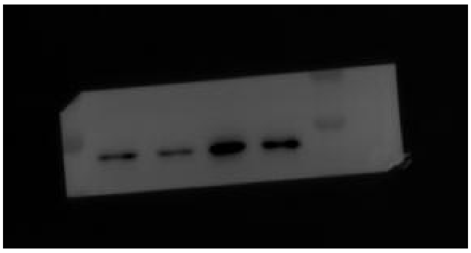

HDAC3

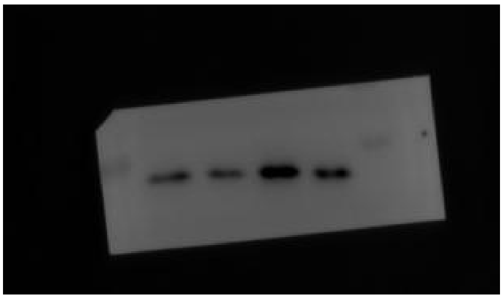
RAB5A

Fig4-D

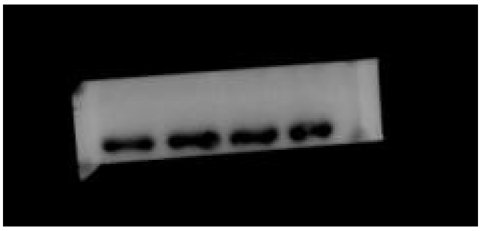
GAPDH

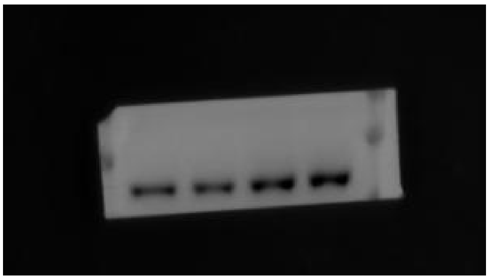
HDAC3

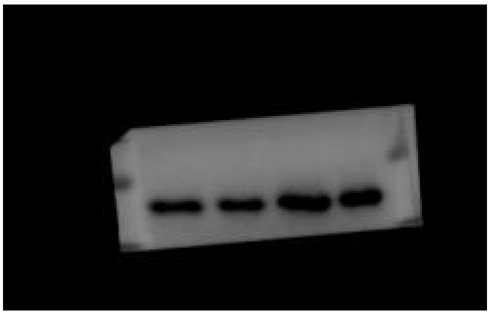
RAB5A

Fig4-E

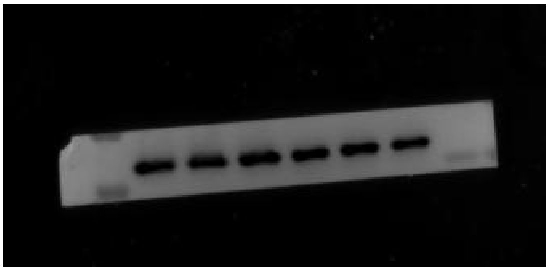
GAPDH

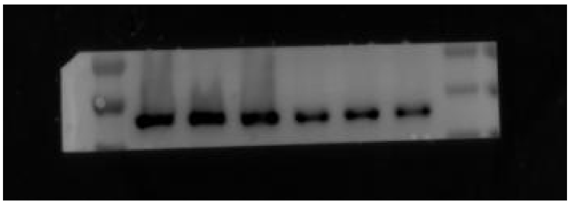

HDAC3

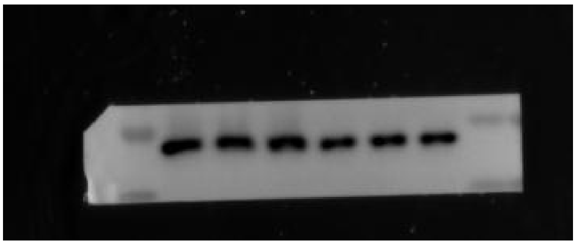

RAB5A

Fig4-F

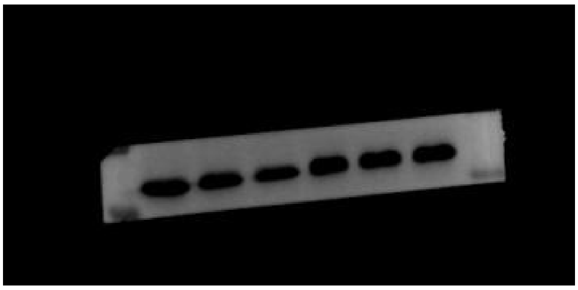

GAPDH

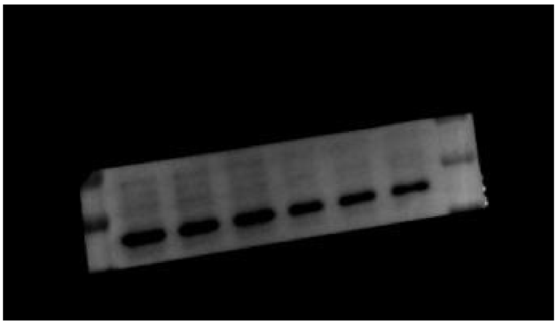

HDAC3

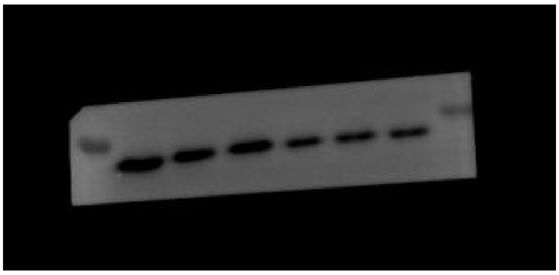

RAB5A

Fig4-J

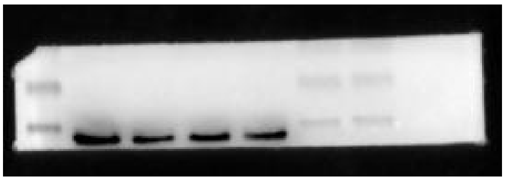

ACTB (P62)

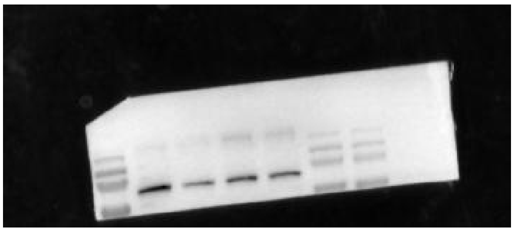

P62

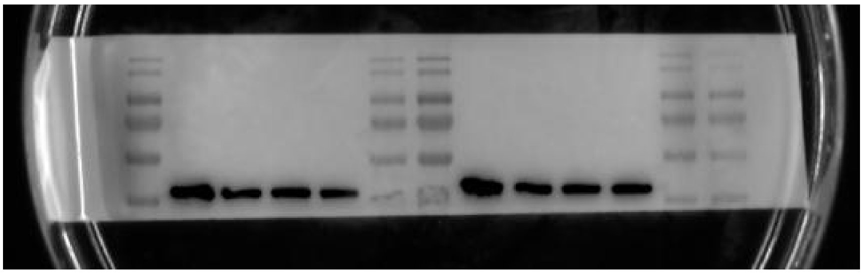

ACTB (LC3B)

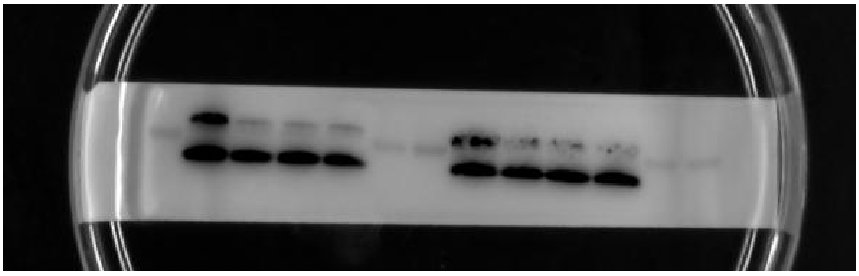

LC3B

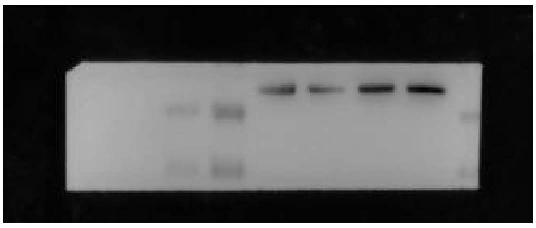

ACTB(RAB5A)

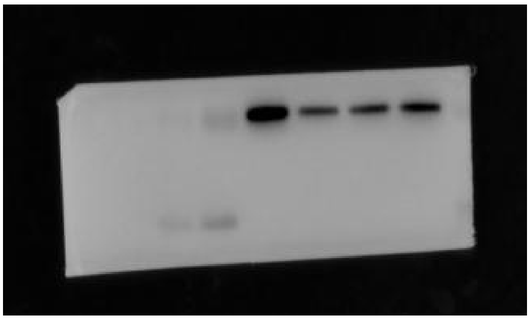

RAB5A

Fig4-K

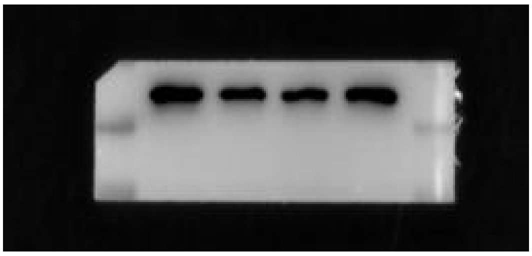

ACTIN

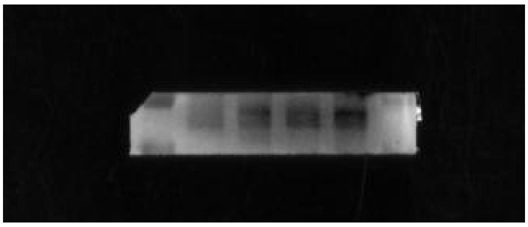

P-AMPK

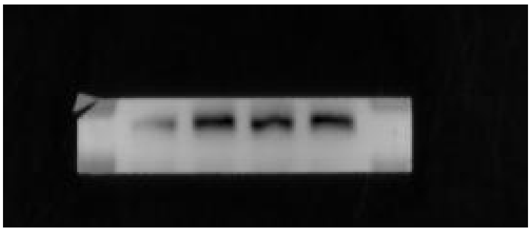

AMPK

Fig4-L

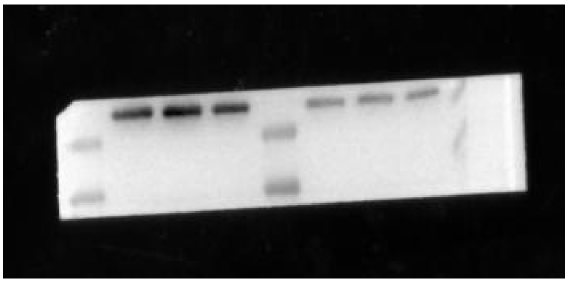

GAPDH

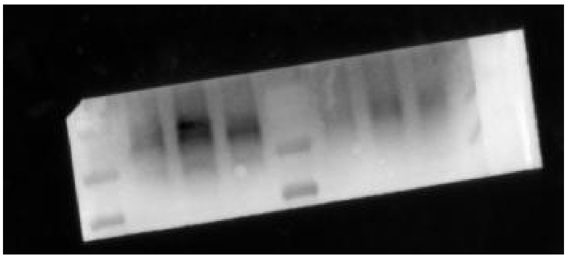

PINK-1

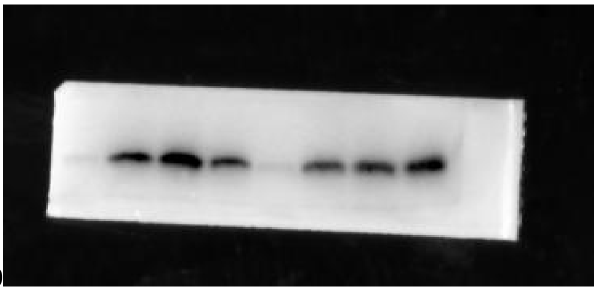

TOMM20

Fig4-M

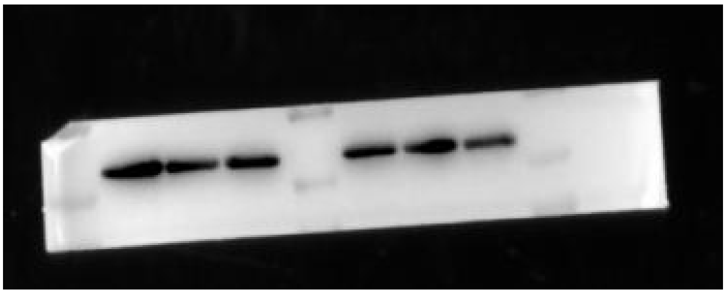

GAPDH

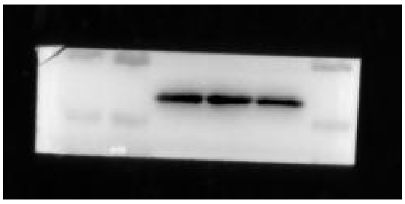

GAPDH

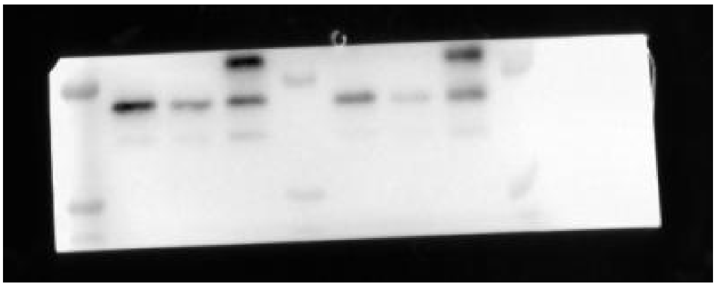

RAB5A

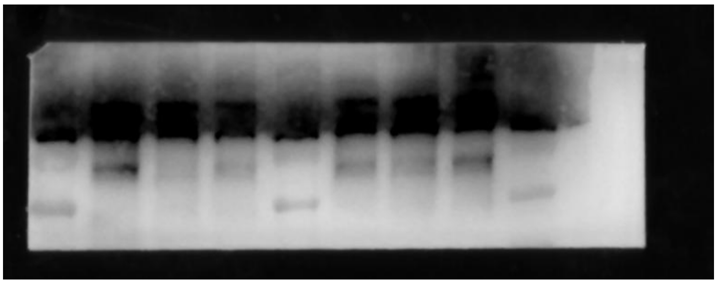

PINK-1

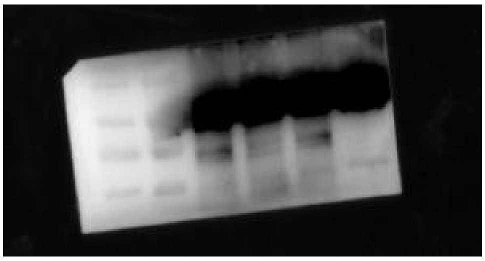

PINK-1

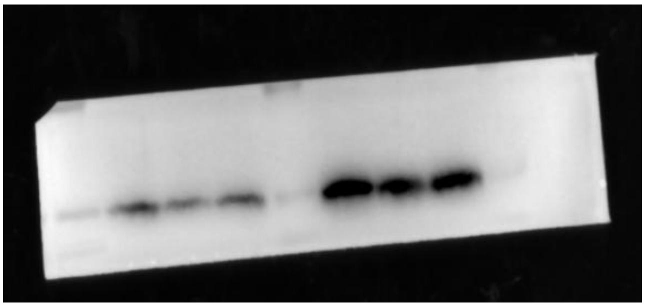

TOMM20

Fig5-C

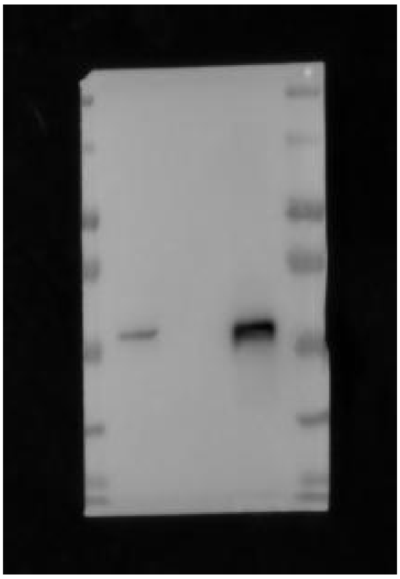
Anti-Flag HDAC3

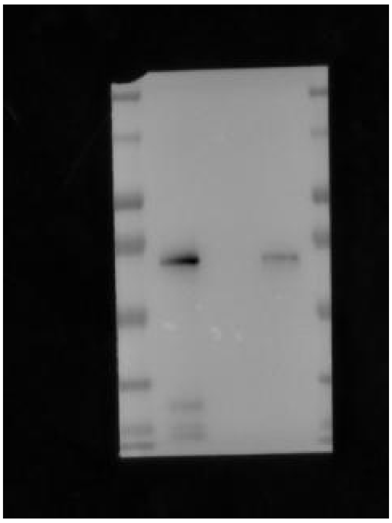
YY1

Fig5-D

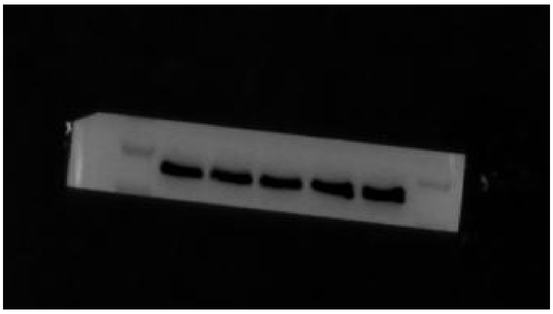
GAPDH

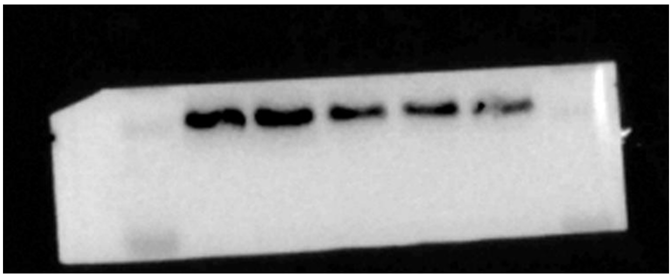

RAB5A

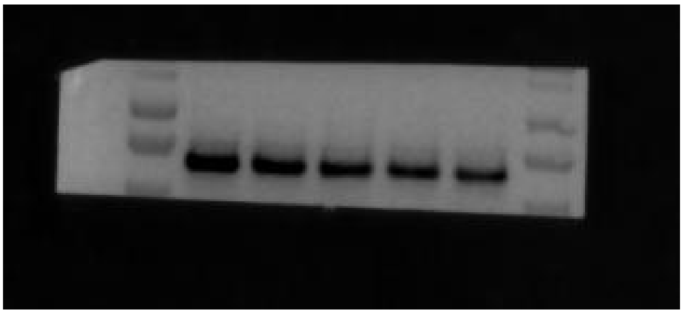

YY1

Fig5-F

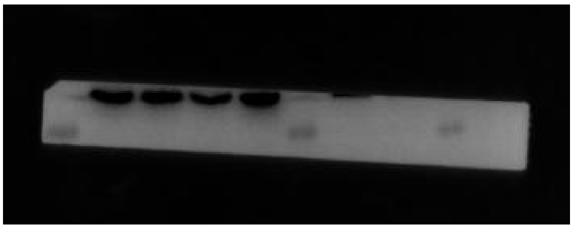

GAPDH

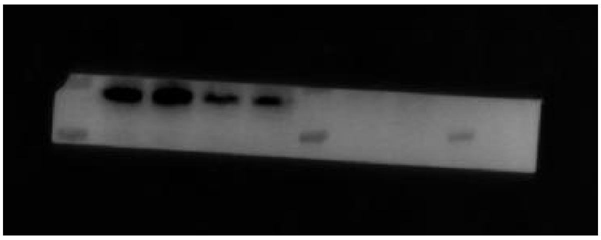

HDAC3

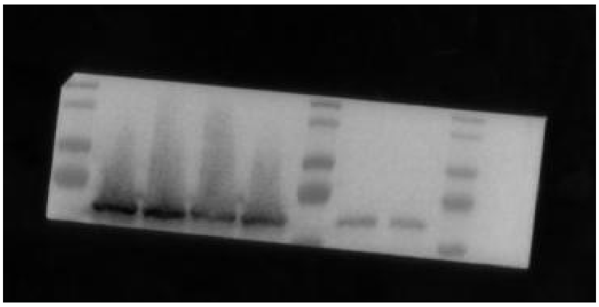

YY1

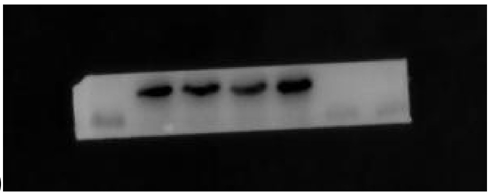

GAPDH (RAB5A)

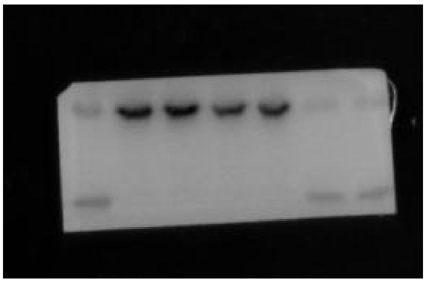

RAB5A

Fig5-G

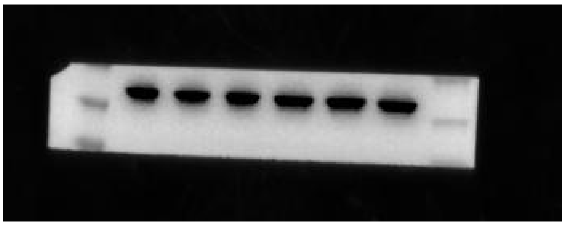

GAPDH

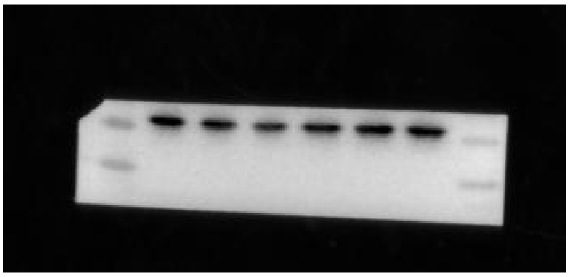

RAB5A

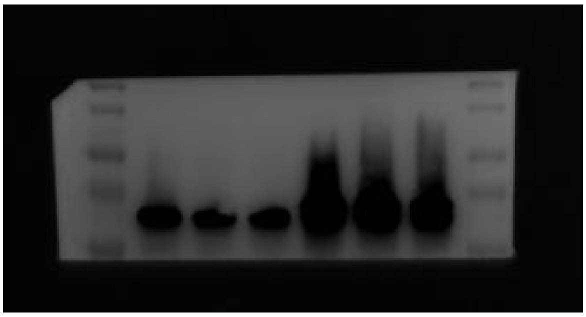

YY1

Fig5-I

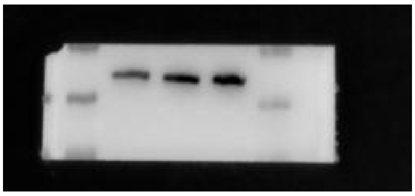

GAPDH

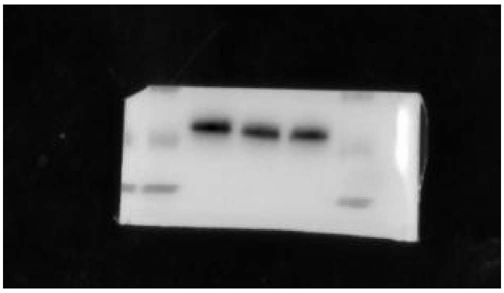
RAB5A

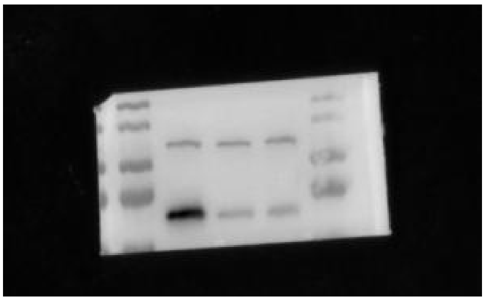
YY1

Fig5-J

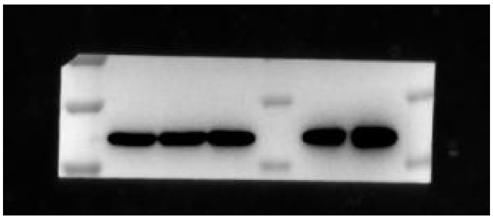
GAPDH

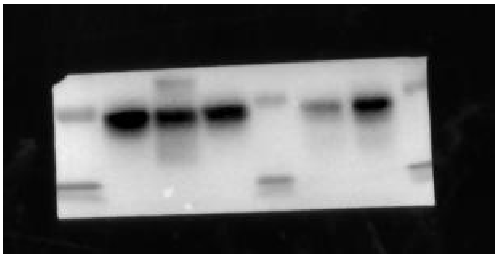
RAB5A

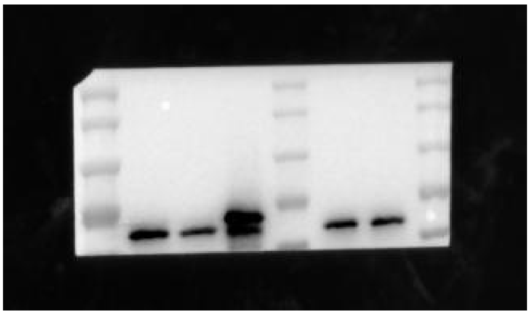
YY1

Fig5-K

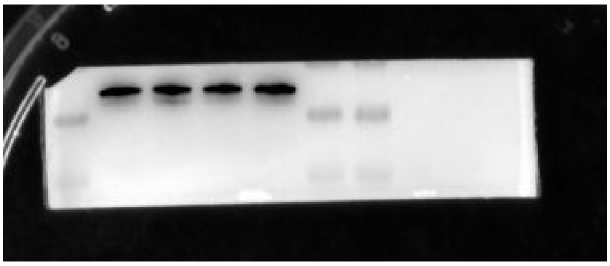

ACTIN

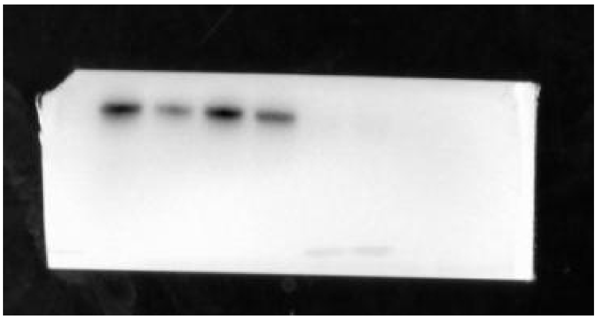

RAB5A

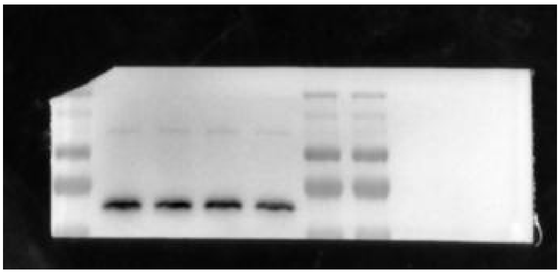

YY1

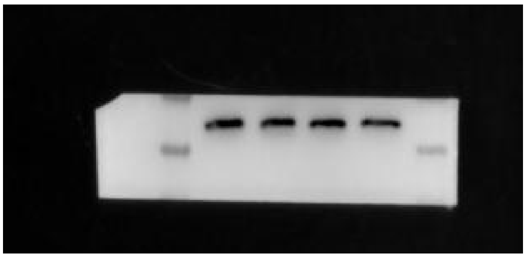

GAPDH

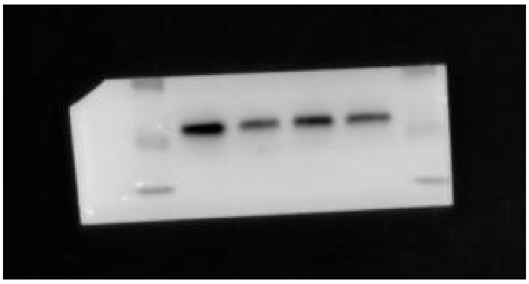

RAB5A

YY1 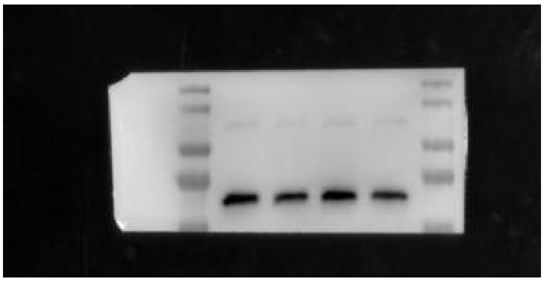

Fig5-M

YY1 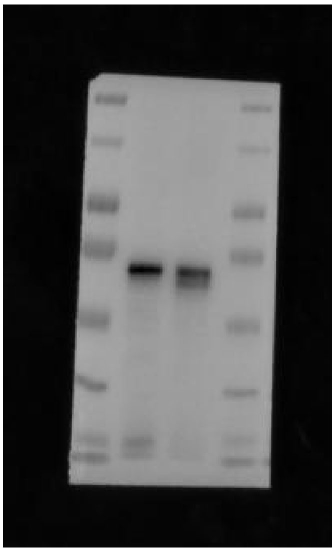 pan-acetylated 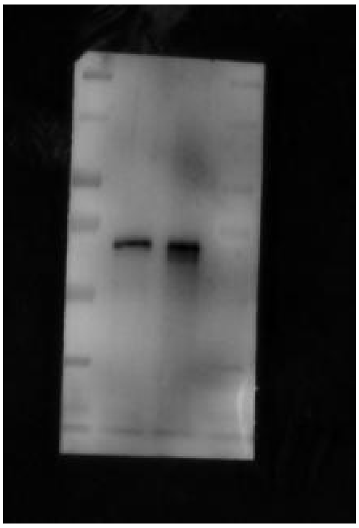

Fig5-N

YY1 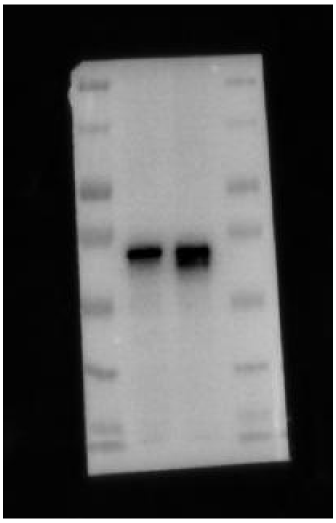 Ub-YY1 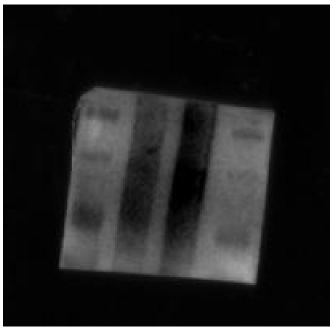

Fig6-A

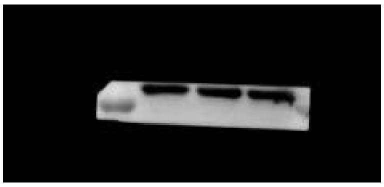

Actin

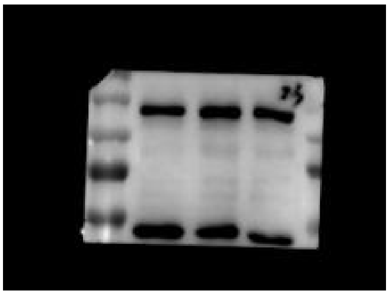

P105/P50

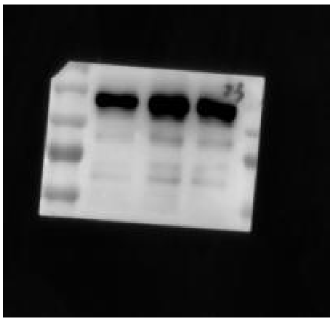

P100/p52

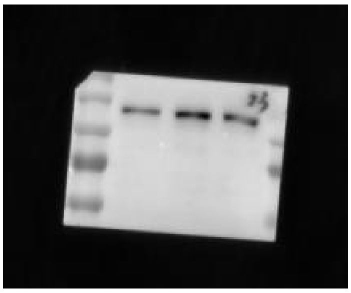

P100/p52

Fig6-D

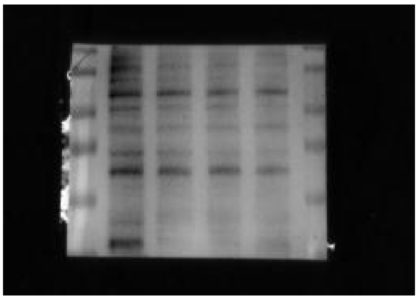

P100/52

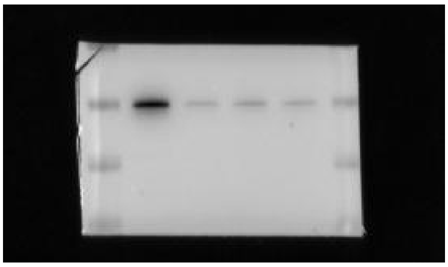

p-ikba

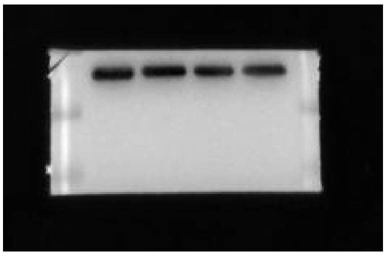

GAPDH

Fig6-E

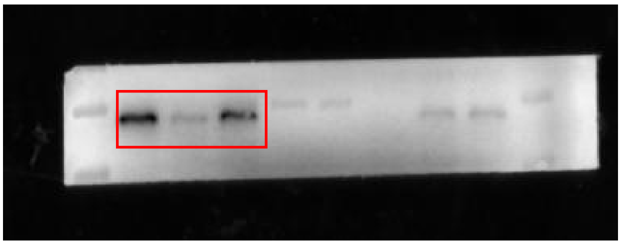

PIKBA

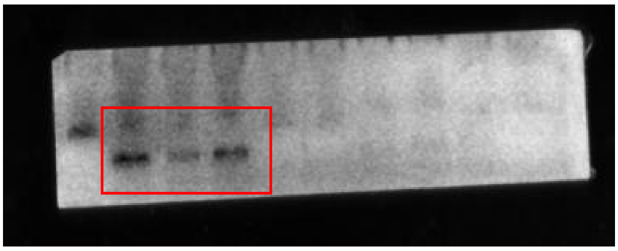

P65

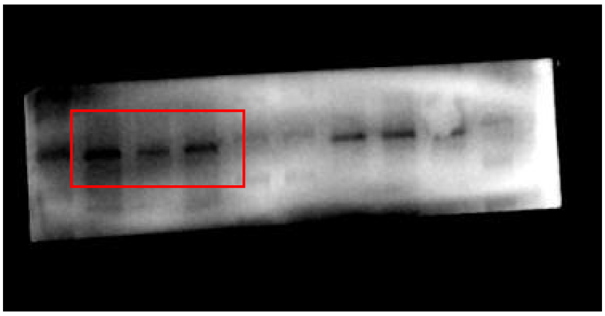

PP65

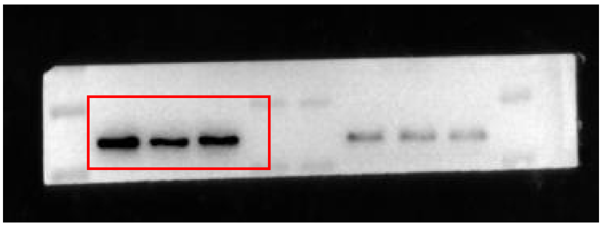

GAPDH

Fig6-F

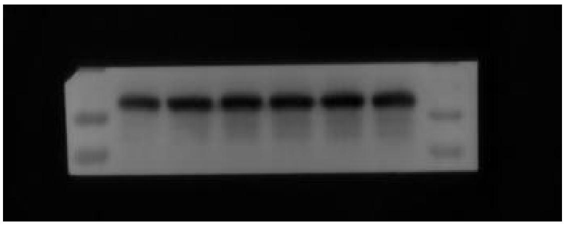

GAPDH

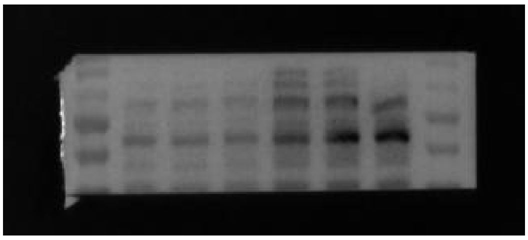

P100/52

Fig6-H

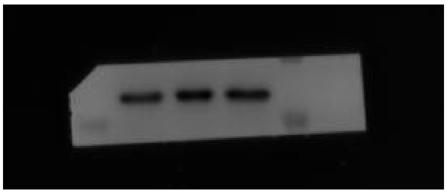

GAPDH

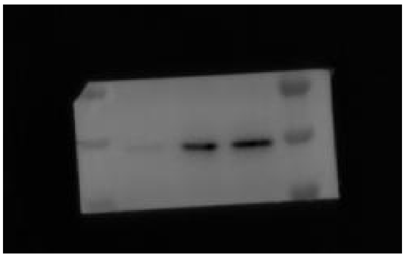

MCL-1

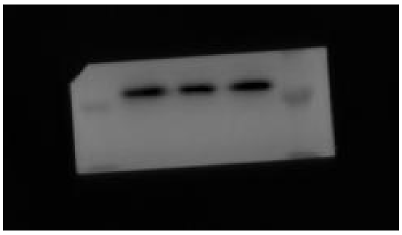

BCL-2

Fig6-I

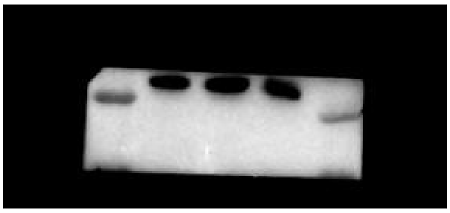

ACTIN

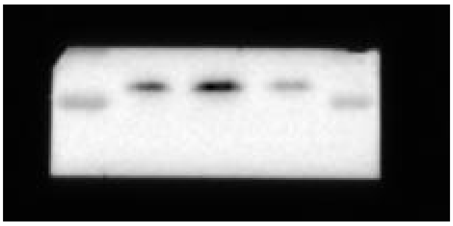

BCL2-

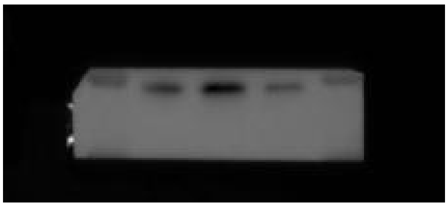

MCL-1

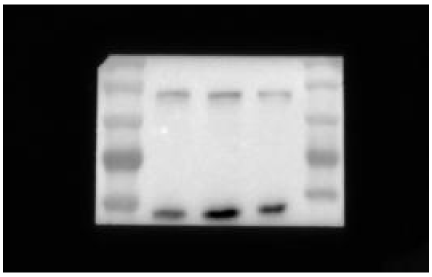

P100/52

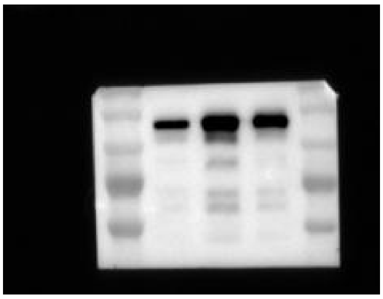

P105/P50

Fig6-J

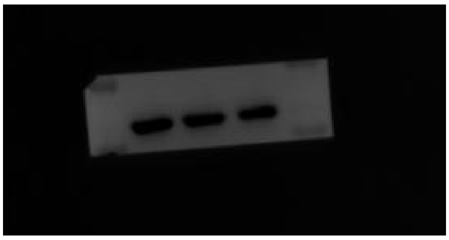
GAPDH

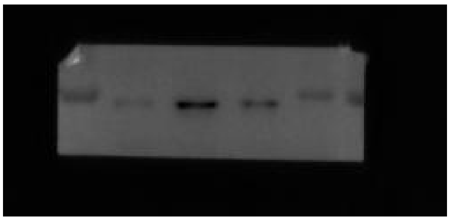
MCL-1

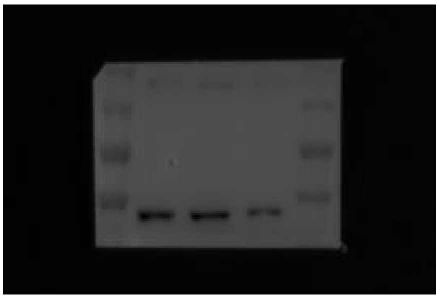
P105/50

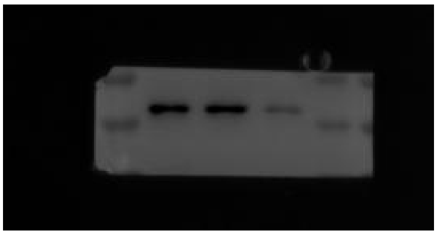
BCL-2

FigS5

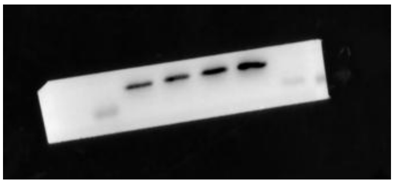
GAPDH

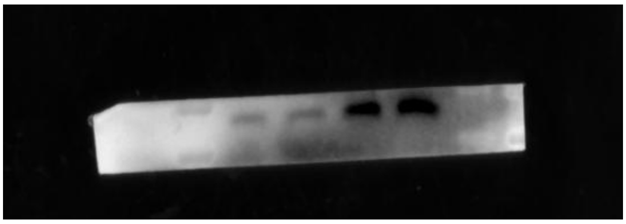
HDAC3

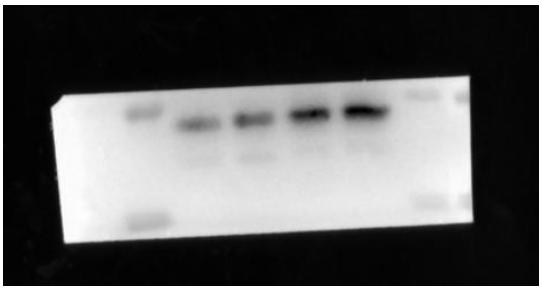

RAB5A

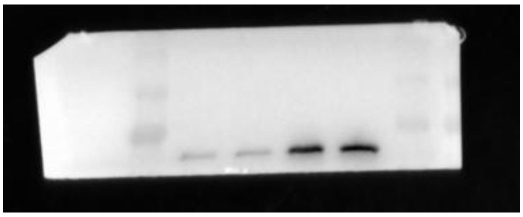

YY1
